# Functional characterization of podocyte-expressed THSD7A in experimental membranous nephropathy

**DOI:** 10.1172/jci.insight.198363

**Published:** 2026-02-26

**Authors:** Ming Huang, Moritz Lassé, Silke Dehde, Felicitas E. Hengel, Fatih Demir, Anja M. Billing, Ning Song, Larissa Seifert, Oliver Kretz, Florian Grahammer, Ulf Panzer, Sebastian Brähler, Tobias B. Huber, Gunther Zahner, Markus M. Rinschen, Nicola M. Tomas

**Affiliations:** 1 III. Department of Medicine, and; 2 Hamburg Center for Kidney Health (HCKH), University Medical Center Hamburg-Eppendorf, Hamburg, Germany.; 3 Department of Biomedicine, Aarhus University, Aarhus, Denmark.; 4 Beijing Anzhen Hospital, Capital Medical University, Beijing, China.; 5 UKE EM Core Facility and; 6 Hamburg Center for Translational Immunology (HCTI), University Medical Center Hamburg-Eppendorf, Hamburg, Germany.; 7 Centre for Molecular Medicine Cologne (CMMC) and; 8 Department II of Internal Medicine, Faculty of Medicine, and University Hospital Cologne, University of Cologne, Cologne, Germany.

**Keywords:** Cell biology, Immunology, Nephrology, Autoimmune diseases, Chronic kidney disease

## Abstract

Although the pathogenic role of autoantibodies targeting the podocyte protein THSD7A in membranous nephropathy (MN) is well described, the consequences of autoantibody binding for podocyte homeostasis and the function of THSD7A remain unclear. Here, we induced an MN model in control and podocyte-specific *Thsd7a*-KO (*Thsd7a^–/–^*) mice using rabbit anti-THSD7A antibodies, followed by transcriptome and proteome analyses. Anti-THSD7A antibodies in WT mice caused significant loss of key slit diaphragm (SD) proteins, such as nephrin and NEPH1, without transcriptional downregulation. Glomeruli showed substantial transcriptomic and proteomic reconfiguration indicative of extensive podocyte injury, including disruptions in podocyte adhesion, cytoskeletal dynamics, and marked upregulation of ubiquitin-proteasome system components, cathepsins, and ADAM proteases. Notably, experiments in C3-deficient mice revealed that proteolytic activation and SD protein loss are driven by complement-independent pathways. *Thsd7a^–/–^* mice only displayed a mild phenotype under basal conditions, and they were completely protected from MN development upon anti-THSD7A antibody transfer. Finally, interactome analysis identified a protein complex, including THSD7A and integrin α3, linking THSD7A complexes to pathogenic regulation of cytoskeleton, adhesion, and membrane signaling in MN. Thus, anti-THSD7A antibodies induce profound molecular reconfiguration, including dysregulated proteolytic systems via a complement-independent pathway, revealing potential therapeutic targets in MN.

## Abstract 2

This study elucidates the action of anti-THSD7A autoantibodies in the kidney in the context of autoimmunity.

## Graphical abstract

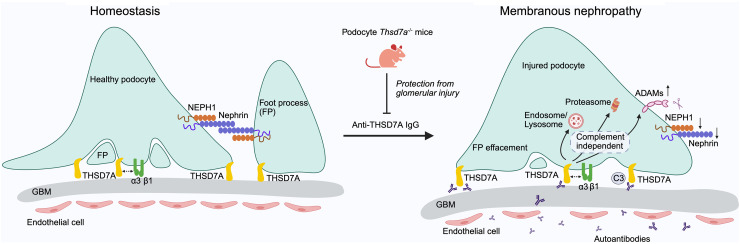

## Introduction

Membranous nephropathy (MN) is an autoimmune kidney disease and the leading cause of nephrotic syndrome in adults. Circulating autoantibodies against podocyte antigens — primarily phospholipase A2 receptor 1 (PLA2R1, ~70% of cases) and thrombospondin type 1 domain-containing protein 7A (THSD7A, ~5%) — bind to targets at the podocyte membrane, triggering immune complex deposition, podocyte injury, and proteinuria (1–3).

THSD7A is a 250 kDa transmembrane protein composed of 21 thrombospondin type 1 (TSP-1) domains, a single transmembrane domain, and a short intracellular tail (4). It localizes to the basal surface of podocyte foot processes, closely associated with the slit diaphragm (SD) (5). This positioning suggests potential involvement of THSD7A in SD regulation and interactions with extracellular matrix proteins in the glomerular basement membrane (GBM). Indeed, THSD7A has been implicated in cell adhesion, cytoskeletal remodeling, and migration in podocytes and endothelial cells (5, 6). In silico modeling also predicts TSP-1 domains of THSD7A as glycosaminoglycan-binding sites for heparan sulfate, a key GBM component, or similar proteoglycans (7).

Substantial progress has been made in identifying MN-associated autoantigens (3, 8–10), but the physiological roles of these proteins and the mechanisms of antibody-mediated injury remain poorly understood. Although complement activation is thought to contribute to podocyte damage, MN models using THSD7A- and PLA2R1-specific antibodies show substantial proteinuria in complement-deficient mice also (11, 12), suggesting complement-independent injury pathways. These may involve disrupted podocyte signaling or altered cytoskeletal regulation (13).

The SD is a specialized intercellular contact point of podocytes, integrating the structural components of multiple cell junction types such as tight, adhesion, gap, and neuronal junctions (14). Core SD proteins, such as nephrin (NPHS1), NEPH1 (KIRREL1), podocin (NPHS2), and CD2-associated protein (CD2AP), coordinate macromolecular filtration, cytoskeletal anchoring, and dynamic signaling in podocytes. However, how SD signaling interfaces with MN pathogenesis remains unclear.

To elucidate the role of THSD7A in glomerular homeostasis and autoantibody-mediated injury, we performed multiomics profiling in a THSD7A-associated MN model and a podocyte-specific *Thsd7a*-KO (*Thsd7a^–/–^*) mouse line. Using these approaches, we identified dysregulated protein degradation pathways as potential mediators of SD protein loss in MN via a complement-independent mechanism. By resolving the distinct roles of THSD7A in homeostasis and disease, this work advances our understanding of antibody-driven podocyte injury and identifies candidate therapeutic targets for MN.

## Results

### Induction of MN by anti-THSD7A IgG leads to glomerular injury and cellular rearrangement.

**Figure 1:**
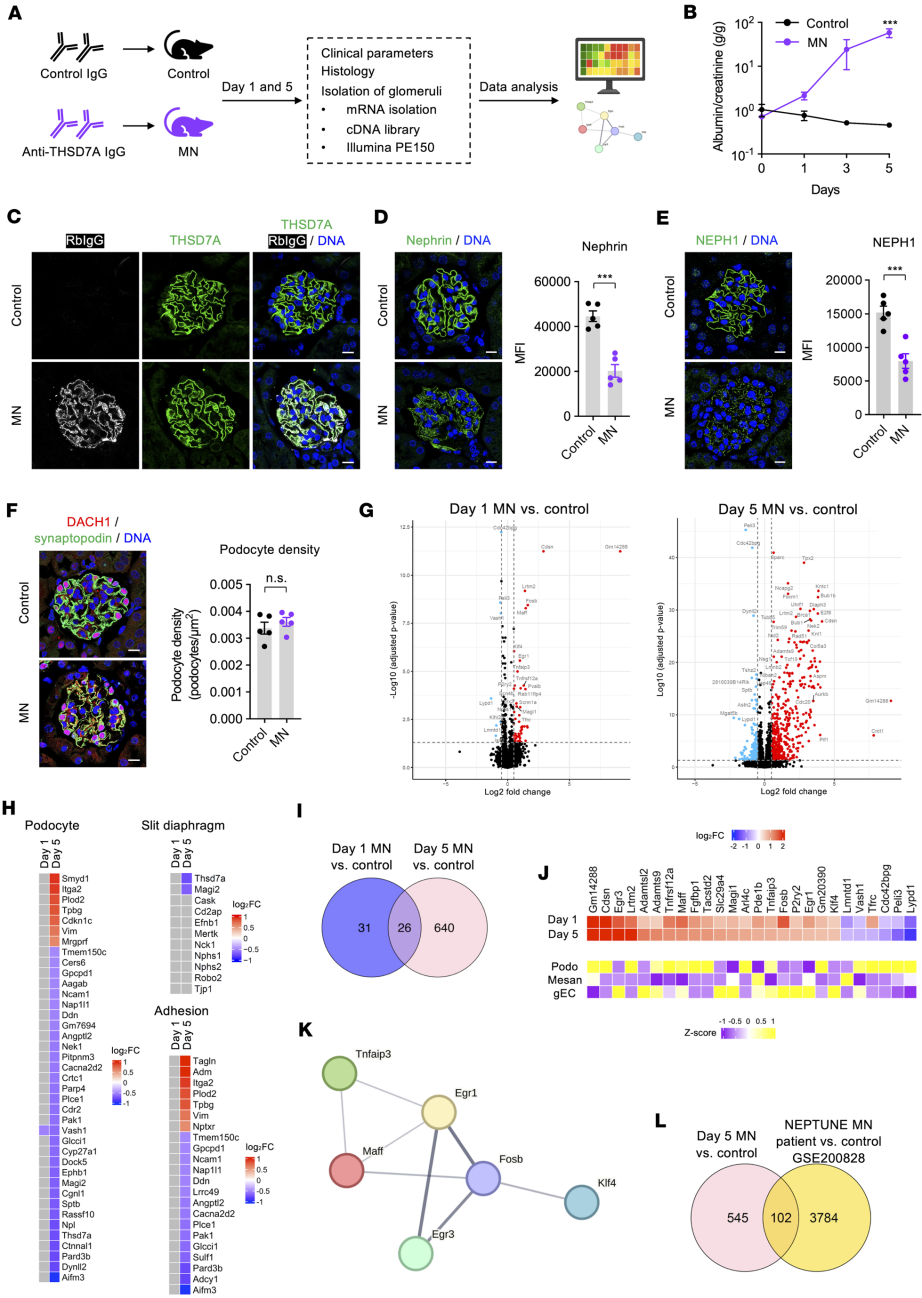
Temporal transcriptomic changes in experimental THSD7A-associated MN. (**A**) Workflow for histology and bulk RNA-Seq in control IgG– and anti-THSD7A IgG–injected mice. (**B**) Urinary albumin-to-creatinine ratio (g/g) over time. ***P < 0.001 (mixed-effects analysis with Bonferroni’s correction). Data are mean ± SEM, *n* = 8–12 per group. (**C**) Representative immunofluorescence staining of rabbit IgG (white) and THSD7A (green) at day 5. Scale bar: 10 μm. (**D**–**F**) Immunofluorescence for nephrin (**D**), NEPH1 (**E**), and DACH1/synaptopodin (**F**) in control and MN mice. Nuclei were counterstained with Hoechst (blue). Scale bar: 10 μm. Right panels: Quantification (MFI) of nephrin and NEPH1, and DACH1-positive podocytes per glomerular tuft. Data are mean ± SEM, *n* = 5. ***P < 0.001 (unpaired 2-tailed *t* test); n.s., not significant. (**G**) Volcano plot of bulk RNA-Seq differential expression. Differentially expressed genes (DEGs) are defined as Benjamini-Hochberg–adjusted *P* value less than 0.05 and |log_2_ fold change (FC)| > 0.5 (*n* = 4–5 biologically independent animals per group). Upregulated DEGs are red, downregulated DEGs blue, others black. (**H**) Heatmaps of log_2_FC of DEGs (day 1 and day 5 MN vs. control) for podocyte, slit diaphragm, and cell adhesion markers. Nonsignificant genes are in gray. (**I**) Venn diagram indicating the exclusive and overlapping DEGs of day 1 and day 5 MN versus control. (**J**) Top: heatmap of 26 DEGs shared between day 1 and day 5 (color indicates log_2_FC). Bottom: heatmap showing the relative expression levels (*z* score) of these genes across major glomerular cell types in a single-cell RNA-Seq dataset (21). Podo, podocytes; Mesan, mesangial cells; gEC, glomerular endothelial cell. (**K**) STRING protein-protein interaction network of key common DEGs of day 1 and day 5. (**L**) Venn diagram showing the exclusive and shared DEGs between day 5 MN versus control and patients with MN versus healthy controls (NEPTUNE glomerular microarray, GEO GSE200828).

To investigate mechanisms of antibody-mediated podocyte injury in MN, we employed an experimental THSD7A-associated MN model (Figure 1A) (15). Mice injected with rabbit anti-THSD7A IgG developed progressive albuminuria (Figure 1B), with granular IgG deposits colocalizing with THSD7A along the glomerular filtration barrier (Figure 1C). In contrast, control IgG-injected mice remained unaffected.

Given the role of SD protein loss and cytoskeletal disruption in podocyte injury (12, 16–18), we assessed the expression of key podocyte markers. In MN mice, THSD7A and synaptopodin remained unchanged (Figure 1C and Supplemental Figure 1, A and B; supplemental material available online with this article; https://doi.org/10.1172/jci.insight.198363DS1), but nephrin and NEPH1 were markedly reduced (Figure 1, D and E, and Supplemental Figure 1, C and D). Podocyte number, based on DACH1 staining, was unaltered (Figure 1F), indicating that SD protein downregulation was independent of podocyte loss. Deposition of the central complement component C3 was observed at the glomerular filtration barrier of MN mice but not of controls (Supplemental Figure 1E).

To capture antibody-induced molecular changes, we performed bulk RNA-Seq on isolated glomeruli from control and MN mice at early (day 1, minimal albuminuria) and late (day 5, overt albuminuria) stages (Figure 1, A and B). Principal component analysis (PCA) showed distinct separation of day 5 MN samples, and day 1 samples partially overlapped with controls, suggesting early changes were modest (Supplemental Figure 2A). Differential expression analysis revealed 57 altered transcripts (50 upregulated and 7 downregulated) on day 1 of MN, increasing to 666 (489 upregulated and 177 upregulated) by day 5 (Figure 1G). Pathway analysis indicated upregulation of cell cycle, DNA repair, extracellular matrix–receptor interaction, and protein kinase signaling in day 5 MN mice (Supplemental Figure 2B). Podocyte-specific transcripts remained stable at day 1 but were mostly downregulated at day 5 (Figure 1H), including 2 SD transcripts, *Magi2* and *Thsd7a*. Genes linked to podocyte adhesion, actin binding, and cytoskeletal remodeling were also dysregulated at day 5 (Figure 1H and Supplemental Figure 2C), reflecting antibody-induced podocyte injury (19, 20).

Across time points, 26 genes were consistently dysregulated (Figure 1, I and J), with more than half exhibiting higher expression in podocytes (21). Pathway enrichment analysis implicated inflammation and cellular stress, such as NGF-NTRK1 signaling, receptor tyrosine kinase activity, and O-glycosylation of thrombospondin type 1 repeat-containing proteins (Supplemental Figure 2D).

Protein network analysis via STRING (22) identified key hubs such as *Tnfaip3* (23), a regulator of NF-κB signaling, and transcription factors *Egr1*, *Egr3*, *Maff*, *Fosb*, and *Klf4* (Figure 1K), all previously implicated in cellular stress responses and injury pathways, including demonstrated roles in podocytes (24–27). We validated key genes by qPCR, which was in line with RNA-Seq findings (Supplemental Figure 2E).

To assess translational relevance, we mapped murine differentially expressed genes to glomerular transcriptomes from patients with MN in the NEPTUNE cohort. We identified 102 conserved transcripts with mostly consistent regulation patterns across species (Figure 1L and Supplemental Figure 3A), and approximately half showed high podocyte expression within glomerulus (Supplemental Figure 3B).

Together, these results define the transcriptomic landscape of anti-THSD7A IgG–induced MN, implicating pathways related to cellular stress, structural disassembly, and inflammation, with relevance to human disease.

### Generation and characterization of podocyte-specific Thsd7a-KO mice.

**Figure 2:**
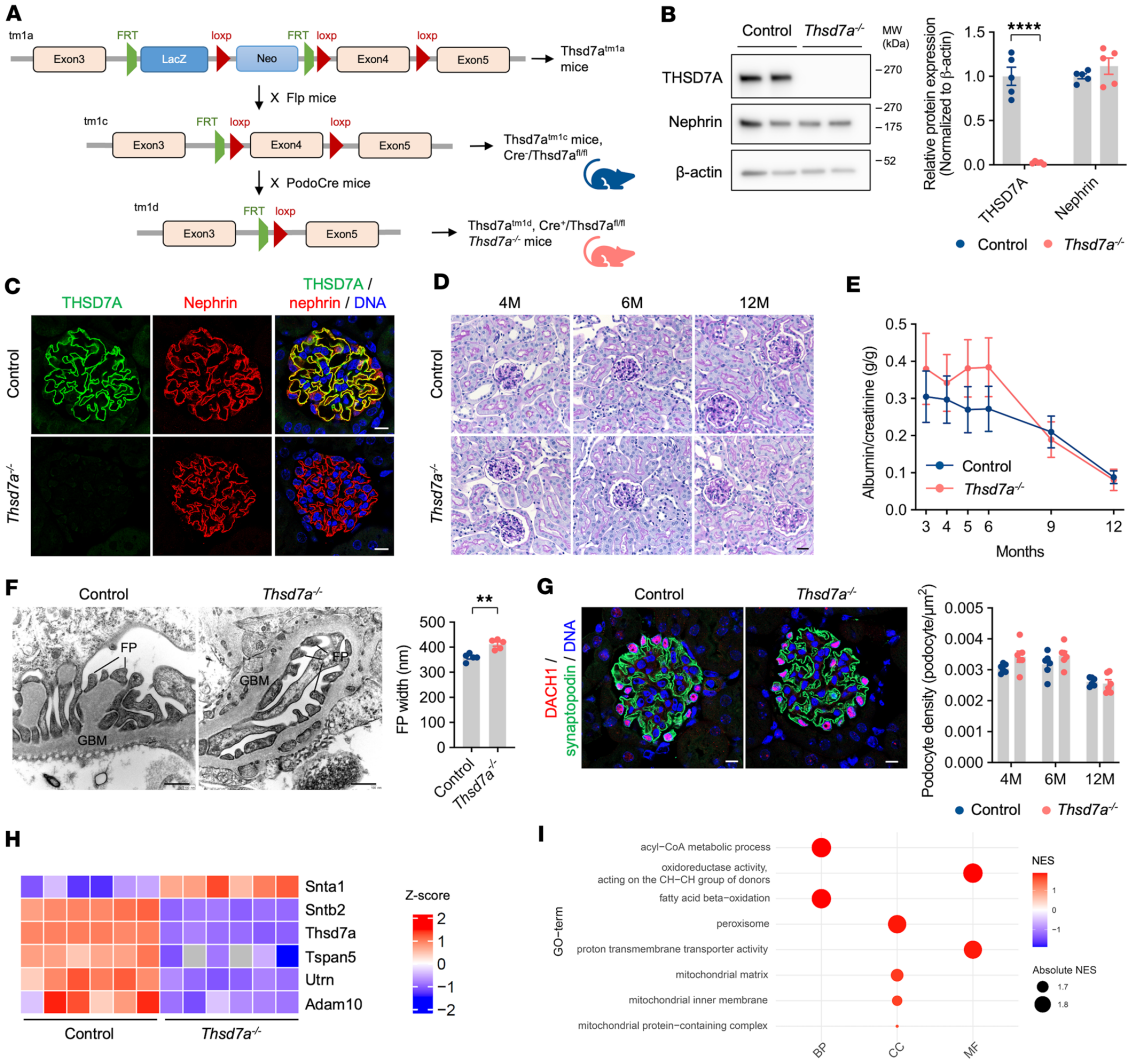
Generation and characterization of podocyte-specific *Thsd7a^–/–^* mice. (**A**) Schematic of generation of podocyte-specific *Thsd7a^–/–^* mice using KO-first strategy. *Thsd7a^tm1c^* mice (Cre^–^/*Thsd7a^fl/fl^*) used as control. (**B**) Representative immunoblotting images revealing decreased expression of THSD7A in isolated glomeruli of *Thsd7a^–/–^* mice (left panel). Individual protein intensity quantified by densitometry and normalized to intensity of β-actin (right panel). ****P < 0.0001 (unpaired 2-tailed *t* test). Data presented as mean ± SEM, *n* = 5. (**C**) Podocyte-specific deletion of THSD7A confirmed by immunofluorescence staining in *Thsd7a^–/–^* mice, and nephrin (red) used as a podocyte marker. Hoechst used as nuclear stain (blue). Scale bar: 10 μm. (**D**) PAS staining of kidney sections showing typical glomerular structures in control and *Thsd7a^–/–^* mice over time. Scale bar: 20 μm. (**E**) Albuminuria measured by albumin-to-creatinine ratio (g/g) over time in control and *Thsd7a^–/–^* mice. Data presented as mean ± SEM, *n* = 22–23 per group. (**F**) Electron microscopic analysis exhibiting glomerular filtration barrier ultrastructure in 6-month-old control and *Thsd7a^–/–^* mice (left panel) and quantification of foot process (FP) width (right panel). GBM, glomerular basement membrane. Scale bar: 500 nm. **P <0.01 (unpaired 2-tailed *t* test). Data presented as mean ± SEM, *n* = 5 per group. (**G**) Representative immunofluorescence staining showing DACH1-positive podocytes (left panel) at 4 months of age and quantification of DACH1-positive podocyte number per glomerular tuft area (right panel). Nuclei counterstained with Hoechst (blue). Scale bar: 10 μm. Data presented as mean ± SEM, *n* = 6 per group. (**H**) Heatmap of row-standardized protein expression (*z* scores of normalized intensities) showing key podocyte markers in control and *Thsd7a^–/–^* mice, measured by mass spectrometry. (**I**) GO enrichment analysis revealing significantly regulated biological processes (BP), cellular components (CC), and molecular functions (MF) in *Thsd7a^–/–^* mice using ClusterProfiler (adjusted *P* < 0.05).

To investigate the role of THSD7A in anti-THSD7A IgG–induced transcriptional changes, we next generated podocyte-specific *Thsd7a*-KO (*Thsd7a^–/–^*) mice. By crossing *Thsd7a^tm1c^* (Cre^–^/*Thsd7a^fl/fl^*) mice with podocin-Cre mice, *Thsd7a^tm1d^* mice (Cre^+^/*Thsd7a^fl/fl^* mice) were established with efficient deletion of exon 4 of *Thsd7a* and selective THSD7A loss in podocytes (Figure 2A). Cre^–^/*Thsd7a^fl/fl^* littermates, which possess a functionally WT *Thsd7a* locus, served as controls. Immunoblot analysis confirmed the absence of THSD7A protein in isolated glomeruli of *Thsd7a^–/–^* mice, with nephrin levels remaining unchanged (Figure 2B). Immunofluorescence showed THSD7A expression adjacent to nephrin in control animal podocytes, whereas THSD7A was absent in *Thsd7a^–/–^* mice (Figure 2C). Analysis across organs revealed highest THSD7A abundance in the kidneys and brain, with lower levels in the heart, lung, spleen, colon, and intestine, consistent with human protein atlas data (28, 29) (Supplemental Figure 4A). THSD7A protein levels remained unchanged in other organs and tubular cells of *Thsd7a^–/–^* mice, confirming KO specificity to podocytes (Supplemental Figures 4, A and B) (30).

Phenotypically, *Thsd7a^–/–^* mice monitored for 12 months showed no major glomerular alterations by light microscopy compared with controls (Figure 2D). *Thsd7a^–/–^* mice, but not control mice and podocin-Cre mice, exhibited a trend toward increased albuminuria during the first 6 months of life (Figure 2E and Supplemental Figure 4C) and a moderate but significant broadening of podocyte foot processes at 6 months (Figure 2F). Although podocyte density declined with age, no significant differences were found between *Thsd7a^–/–^* and control mice, as indicated by DACH1 staining (Figure 2G). These results suggest that podocyte-specific THSD7A deficiency mildly compromises the glomerular filtration barrier without causing severe damage up to 12 months of age.

### Loss of Thsd7a is largely compensated by changes in scaffold proteins and metabolism.

To better understand the mild structural and functional changes in *Thsd7a*^–/–^ mice, we performed proteomic analysis of isolated glomeruli from *Thsd7a*^–/–^ mice and controls. Among 7,247 identified proteins, THSD7A, SNTB2, and TSPAN5 were significantly downregulated in *Thsd7a^–/–^* mice (Supplemental Figure 4D), with THSD7A levels reduced approximately 50-fold in the KO mice. Notably, the syntrophin adaptor proteins SNTA1 and SNTB2, their cytoskeletal anchor utrophin (UTRN), the tetraspanin TSPAN5, and its interacting metalloprotease ADAM10 were markedly dysregulated (Figure 2H). Syntrophins function as scaffold proteins linking surface receptors, ion channels, and signaling proteins to dystrophin/utrophin complexes, thereby mediating signal transduction and critical cellular processes (31). TSPAN5, a tetraspanin of the C8 subgroup, regulates intracellular trafficking and ADAM10 activity (32), which was also downregulated in *Thsd7a^–/–^* glomeruli. Key SD-associated proteins remained unchanged (highlighted in orange in Supplemental Figure 4D). These findings suggest that THSD7A deficiency is partly compensated by alterations in scaffolding and proteolytic proteins, helping maintain physiological function in podocytes.

Gene set enrichment analysis revealed that proteins involved in metabolic pathways — such as acyl-CoA metabolism, fatty acid beta-oxidation, proton transmembrane transport, and oxidoreductase activity — were enriched in *Thsd7a^–/–^* glomeruli (Figure 2I). This enrichment indicates increased energy demand in the absence of THSD7A, potentially compensated by enhanced lipid and glucose metabolism. Overall, these data suggest that loss of THSD7A in podocytes triggers compositional changes in membrane proteins alongside elevated metabolic energy expenditure.

### Thsd7a^–/–^ mice are protected from proteinuria and podocyte damage in experimental THSD7A-associated MN.

**Figure 3:**
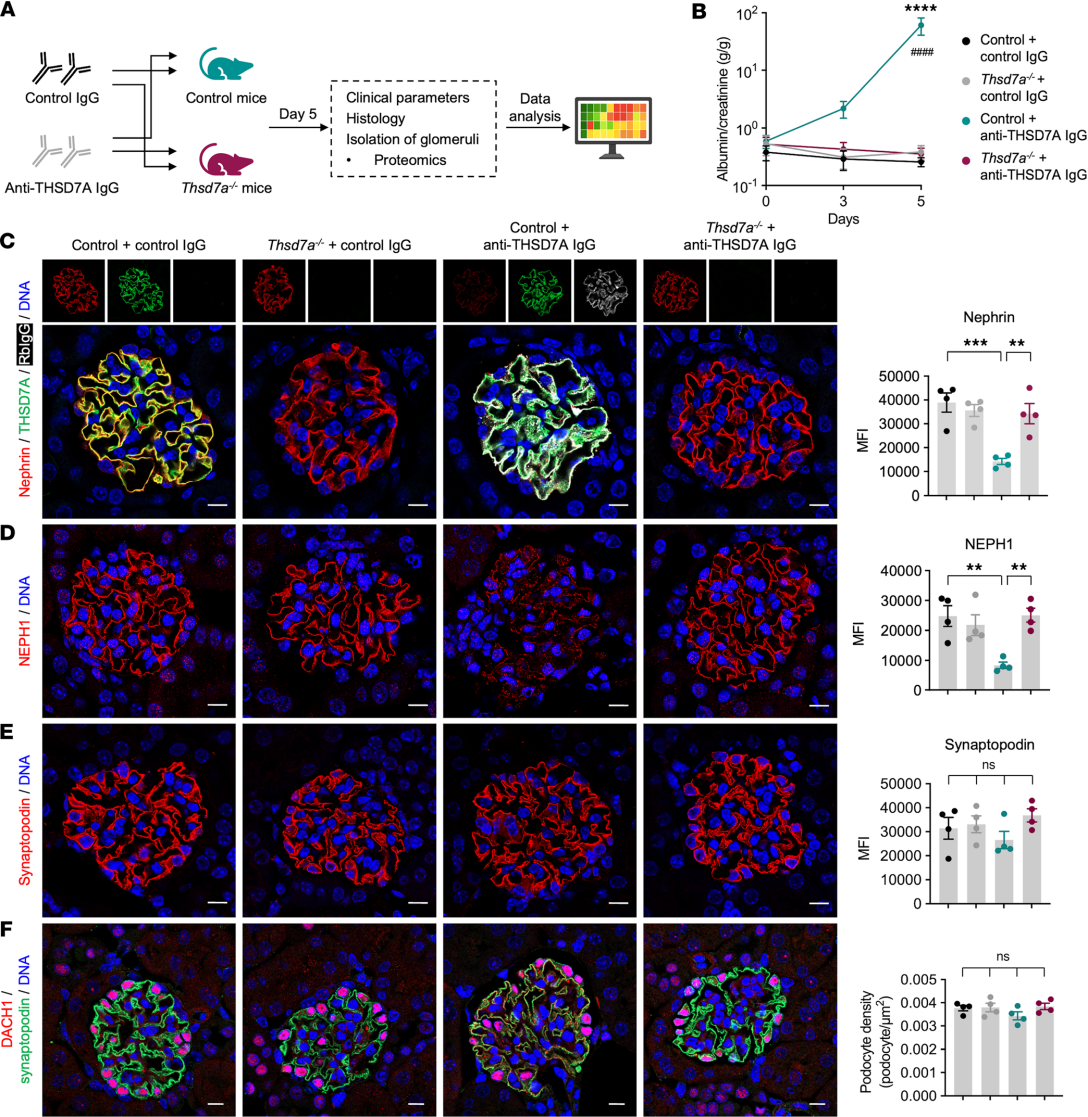
Podocyte-specific *Thsd7a^–/–^* mice are protected from antibody-induced podocyte injury. (**A**) Experimental workflow for control and *Thsd7a^–/–^* mice injected with control IgG and anti-THSD7A IgG. (**B**) Albumin-to-creatinine ratio (g/g) over time. ****P < 0.0001 (control mice + anti-THSD7A IgG vs. control mice + control IgG), ^####^*P* < 0.0001 (control mice + anti-THSD7A IgG vs. *Thsd7a^–/–^* mice + anti-THSD7A IgG) (2-way ANOVA with Bonferroni’s multiple-comparison test). Data shown as mean ± SEM, *n* = 7–8 per group. (**C**) Left: immunofluorescence stainings for rabbit IgG, nephrin, and THSD7A 5 days after injection of control IgG or anti-THSD7A IgG (left panel). Scale bars: 10 μm. Right: quantification (MFI) of nephrin. **P < 0.01, ***P < 0.001 (1-way ANOVA with Tukey’s multiple-comparison test). Data shown as mean ± SEM, *n* = 4 per group. (**D** and **E**) Left: immunofluorescence stainings for NEPH1 (**D**, left panel) and synaptopodin (**E**, left panel). Scale bars: 10 μm. Right: quantification (MFI) of NEPH1 (**D**) and synaptopodin (**E**). **P < 0.01 (1-way ANOVA with Tukey’s multiple-comparison test). Data shown as mean ± SEM, *n* = 4 per group. (**F**) Left: immunofluorescence stainings showing DACH-1–positive podocytes. Scale bar: 10 μm. Right: quantification of podocyte number per glomerular tuft area. Data shown as mean ± SEM, *n* = 4 per group analyzed (1-way ANOVA with Tukey’s multiple-comparison test); n.s., not significant.

We next investigated whether podocyte-specific deletion of THSD7A confers protection against anti-THSD7A antibody–mediated glomerular injury. To test this question, both *Thsd7a^–/–^* mice and controls were injected with either control IgG or anti-THSD7A rabbit IgG (Figure 3A). Injection of anti-THSD7A IgG induced significant albuminuria in controls, whereas *Thsd7a^–/–^* mice were completely protected from albuminuria (Figure 3B). Immunofluorescence analysis revealed strong rabbit IgG positivity colocalizing with THSD7A along the glomerular filtration barrier in controls treated with anti-THSD7A IgG (Figure 3C). By contrast, no detectable rabbit IgG was observed in *Thsd7a^–/–^* mice injected with anti-THSD7A IgG or in mice receiving control IgG. Notably, nephrin (Figure 3C) and NEPH1 (Figure 3D), but not synaptopodin (Figure 3E), were markedly reduced and appeared scattered in anti-THSD7A IgG–injected controls, indicating severe SD disruption. Immunofluorescence staining for DACH1 showed no significant reduction in podocyte density across all groups (Figure 3F), indicating that the severe decreases in nephrin and NEPH1 reflect protein regulation changes rather than podocyte loss. Together, these findings demonstrate that *Thsd7a^–/–^* mice are protected from MN in the presence of anti-THSD7A antibodies.

### Proteomic analysis of experimental THSD7A-associated MN.

**Figure 4:**
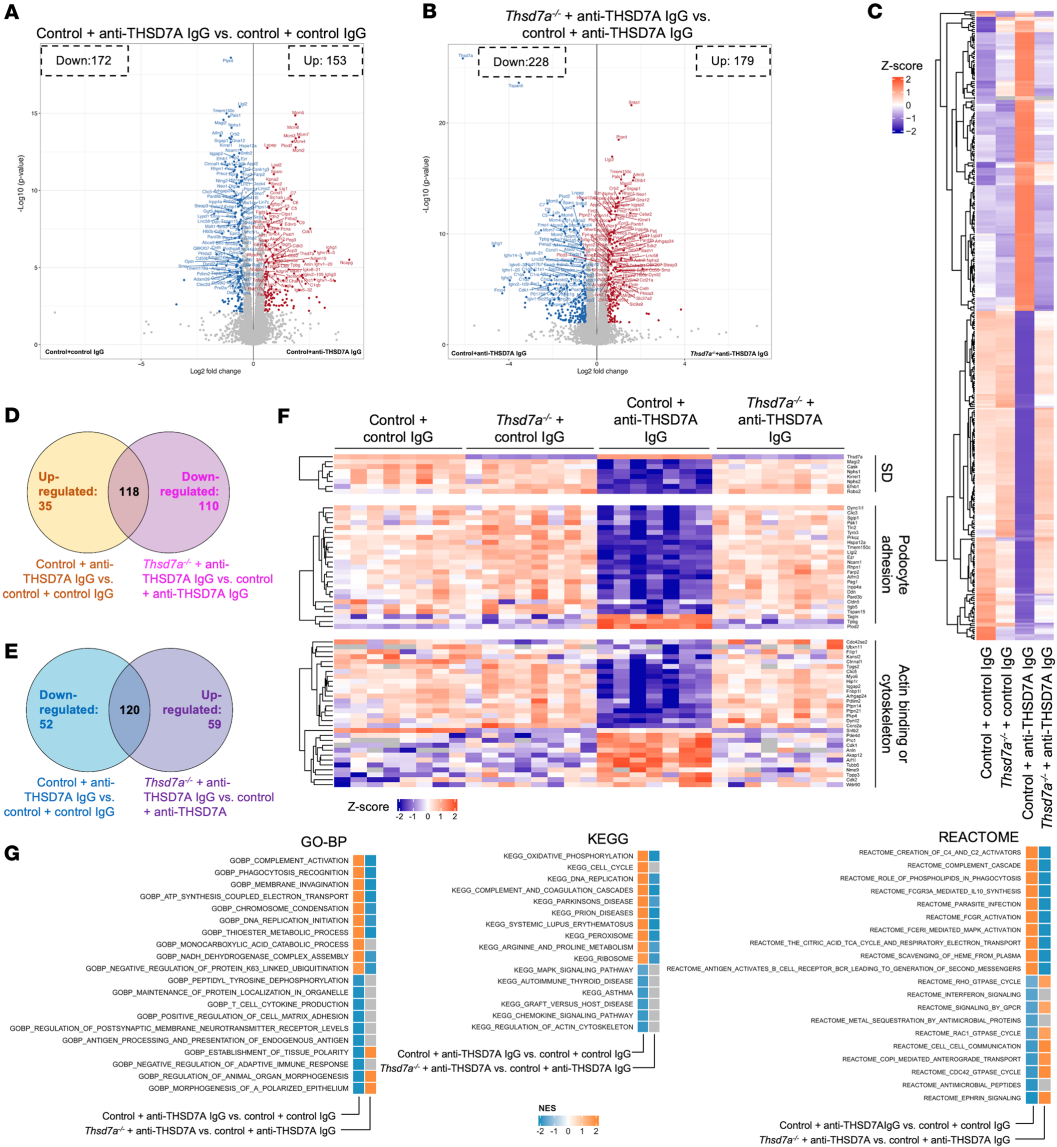
Proteome alterations in experimental THSD7A-associated MN. (**A** and **B**) Volcano plots of the differential expression analysis from proteomic analysis of isolated glomeruli from control mice and *Thsd7a^–/–^* mice treated with control or anti-THSD7A IgG. In **A**, the control + anti-THSD7A IgG group was compared with the control + control IgG group, *n* = 7–8. In **B**, the *Thsd7a^–/–^*+ anti-THSD7A IgG group was compared with the control + anti-THSD7A IgG group, *n* = 7–8. *X* axis represents the log_2_FC of label-free quantification intensity of proteins by comparisons relatively, and *y* axis indicates the -log_10_ *P* value. Proteins marked in red or blue are significantly upregulated or downregulated, respectively (Benjamini-Hochberg–adjusted *P* < 0.05, |log_2_FC| > 0.5). (**C**) Heatmap of protein expression showing row-wise *z* scores of normalized intensities (group means from 7–8 replicates per group) across the 4 treatment groups. The heatmap includes all proteins that were significantly regulated in the control + anti-THSD7A IgG group versus control + control IgG group. (**D**) Venn diagram showing the significant proteins upregulated in control + anti-THSD7A IgG versus control + control IgG groups and downregulated in *Thsd7a^–/–^*+ anti-THSD7A IgG versus control + anti-THSD7A IgG groups. (**E**) Venn diagram showing the significant proteins downregulated in control + anti-THSD7A IgG versus control + control IgG groups and upregulated in *Thsd7a^–/–^* + anti-THSD7A IgG versus control + anti-THSD7A IgG groups. (**F**) Heatmap showing row-standardized protein expression (*z* scores of normalized intensities) of podocyte slit diaphragm proteins, podocyte adhesion proteins, and actin binding or cytoskeleton proteins. (**G**) GO-BP, KEGG, and Reactome enrichment analysis of proteins significantly regulated in comparisons of control + anti-THSD7A IgG versus control + control IgG or *Thsd7a^–/–^* + anti-THSD7A versus control + anti-THSD7A IgG groups, using fgsea (adjusted *P* < 0.05).

To further dissect the mechanisms underlying antibody-mediated injury in THSD7A-associated MN, we performed label-free quantitative data–independent proteomics on isolated glomeruli from experimental mice. In total, we identified 9,364 proteins, and PCA revealed clear separation between treatment groups according to the experimental conditions (Supplemental Figure 5A). Comparing controls injected with anti-THSD7A IgG to controls injected with control IgG, we detected 325 significantly regulated proteins (172 downregulated, 153 upregulated) (Figure 4A). When comparing controls with *Thsd7a^–/–^* mice, both injected with anti-THSD7A IgG, 407 proteins were significantly regulated (228 downregulated, 179 upregulated) (Figure 4B).

Podocyte-specific KO of *Thsd7a* prevented the glomerular damage induced by anti-THSD7A IgG observed in controls (Figure 4C). Integrating the datasets from Figure 4, A and B, we identified 118 proteins consistently upregulated in controls injected with anti-THSD7A IgG but reversed in *Thsd7a^–/–^* mice (Figure 4D and Supplemental Figure 5B). These proteins were primarily involved in complement activation, oxidative stress response, and DNA damage pathways (Supplemental Figure 5B). Conversely, 120 proteins downregulated in anti-THSD7A IgG–injected controls were restored in *Thsd7a^–/–^* mice (Figure 4E and Supplemental Figure 5C), with functions linked to epithelial cell polarity, podocyte development, and cell migration.

Notably, several SD proteins such as MAGI2, nephrin, podocin, and NEPH1, but not THSD7A, were markedly decreased in anti-THSD7A IgG–injected controls, indicating disruption of the functional SD (Figure 4F). In contrast, these SD proteins remained at levels comparable to controls in anti-THSD7A IgG–injected *Thsd7a^–/–^* mice (Figure 4F). Furthermore, antibody binding in control MN animals led to complement activation (Supplemental Figure 5D) and downstream podocyte injury, reflected by changes in key adhesion proteins and actin-binding/cytoskeletal components, which were largely mitigated in *Thsd7a^–/–^* mice (Figure 4F and Supplemental Figure 5E).

Gene set enrichment analysis revealed strong activation of pathways related to complement activation, phagocytosis, DNA replication, and electron transport in MN (Figure 4G). Conversely, pathways associated with epithelial and organ morphogenesis, matrix adhesion regulation, MAPK signaling, RHO GTPase activity, and ephrin signaling were suppressed, suggesting glomerular injury characterized by impaired adhesion and cytoskeletal dynamics (Figure 4G). Importantly, KO of *Thsd7a* completely reversed these pathway alterations (Figure 4G).

Together, these results indicate that anti-THSD7A IgG induces extensive proteomic alterations and glomerular injury in controls, while podocyte-specific *Thsd7a* deletion confers robust protection against these changes.

### Integration of transcriptomic and proteomic analyses reveals protein degradation as key mechanisms in experimental MN.

**Figure 5:**
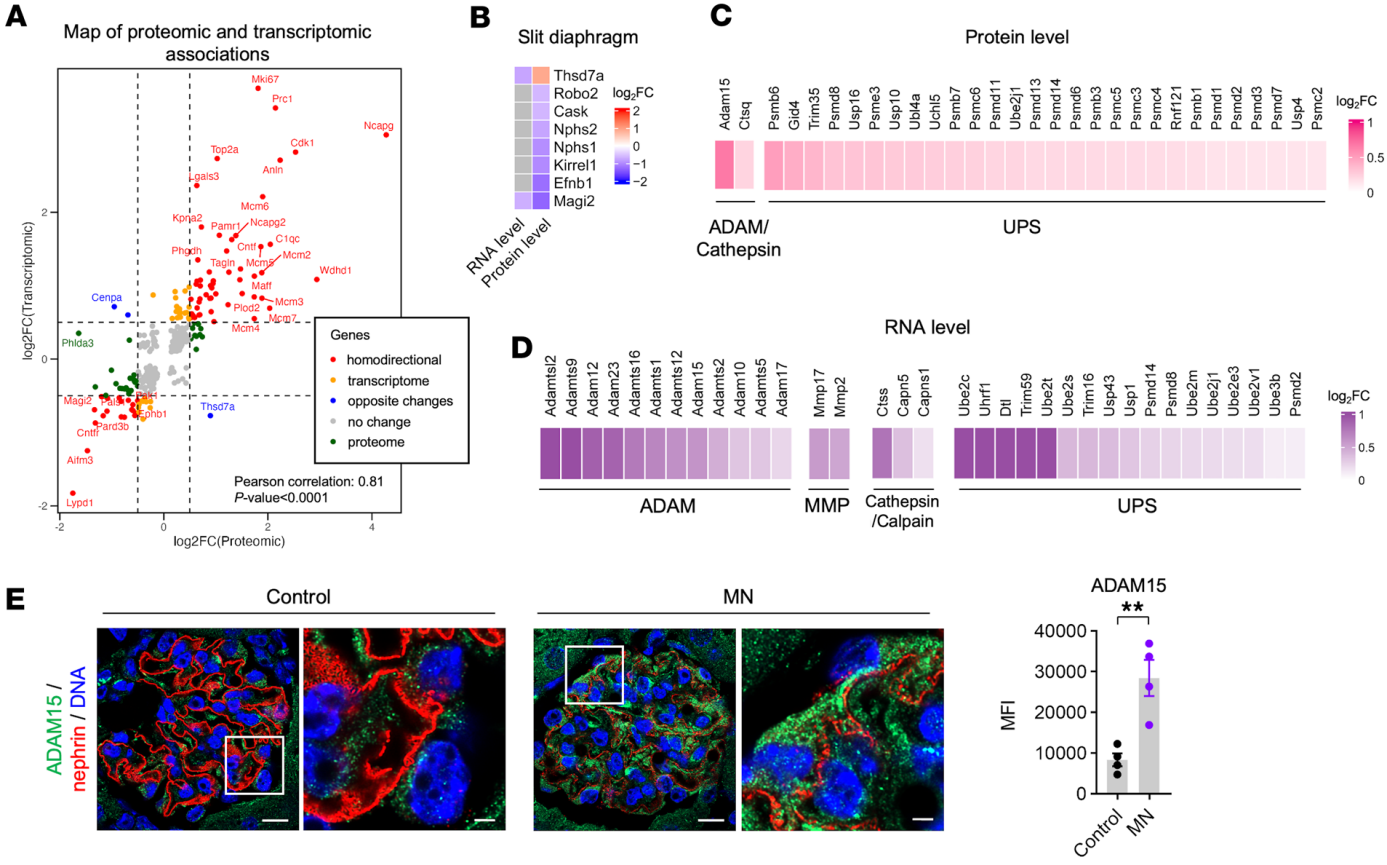
Correlation of transcriptomic and proteomic data reveals protein degradation as a key mechanism in THSD7A-associated MN. (**A**) Correlation between transcriptomic and proteomic gene expression levels in experimental THSD7A-associated MN at day 5. After filtering by adjusted *P* value less than 0.05, the log_2_ FC of genes (day 5 MN vs. control) from RNA-Seq is plotted against the log_2_FC of proteins (control + anti-THSD7A IgG group vs. control + control IgG group). The horizontal and vertical dashed lines indicate the threshold log_2_FC = ± 0.5. Pearson’s correlation and *P* value were calculated. Colored dots correspond to the differentially expressed genes/proteins. In the legend, homodirectional means upregulated or downregulated both in transcriptome and proteome levels; opposite change is upregulated at one level while downregulated at the other; proteome means only significantly regulated in protein levels; transcriptome means only significantly regulated in transcript level. (**B**) Heatmaps declaring the log_2_FC of slit diaphragm genes and proteins in comparison of MN versus control groups. Nonsignificant genes are shown in gray. (**C** and **D**) Heatmaps showing the log_2_FC of protein degradation–associated proteins in proteomic data (**C**) or genes in bulk RNA-Seq data (**D**) in MN versus control comparisons. (**E**) Left: immunofluorescence stainings for ADAM15 (green) and nephrin (red) 5 days after injection of control IgG or anti-THSD7A IgG. Nuclei were counterstained with Hoechst (blue). Scale bars: 10 μm (zoom-out panels); 2 μm (zoom-in panels). Right: quantification (MFI) of ADAM15. **P < 0.01 (unpaired 2-tailed *t* test). Data shown as mean ± SEM, *n* = 4 per group.

To develop a comprehensive understanding of antibody-mediated injury in experimental THSD7A-associated MN, we integrated transcriptomic and proteomic datasets. We identified 276 significantly regulated gene products (transcripts or proteins) common to both datasets, filtered by adjusted *P* value less than 0.05 (Figure 5A). Among these, 53 gene products, including genes related to cytoskeletal remodeling and stress response (*Anln*, *Maff*, *Plod2*, *Tagln*, and *Ednrb*) and genes involved in DNA replication and cell cycle progression (such as *Mcm2-7*, *Wdhd1*, *Top2a*, *Ncapg*, *Ncapg2*, *Cdk1*, *Smc2/4*), were significantly upregulated at both mRNA and protein levels. Conversely, 19 genes, such as *Ephb1*, *Magi2*, *Pak1*, *Pals1*, and *Pard3b* — key players in epithelial cell signaling — were downregulated across both datasets. The high correlation between transcriptomic and proteomic changes (Pearson’s correlation = 0.81, Figure 5A) underscores the robustness and directional consistency of these alterations. Consistent with clinical observations where MN-specific antigens are elevated (33), THSD7A protein levels were increased; interestingly, however, *Thsd7a* transcript levels were decreased, suggesting altered protein translation and/or turnover. The discrepancy between the unchanged THSD7A pattern in immunofluorescence and the increased detection in the proteome analysis may be explained by reduced accessibility of the protein for the staining antibody in immunofluorescence or protein conformational changes as a consequence of the binding of rabbit anti-THSD7A antibodies in the model.

This strong correlation between transcriptomic and proteomic changes extended to enriched pathways (Supplemental Figure 6). DNA replication and cell cycle pathways were predominantly enriched among commonly upregulated gene products in both datasets. In contrast, complement-related pathways were more enriched in the proteomic dataset, indicating the deposition of circulating complement factors rather than local complement production.

A notable discordance emerged regarding key SD components. Significant decreases were observed at the protein level for nephrin, podocin, NEPH1, ephrin B1 (EFNB1), CASK, and ROBO2 (Figure 5B), consistent with immunofluorescence and immunoblot data showing loss of nephrin and NEPH1 in glomeruli (Figure 1, D and E, Supplemental Figure 1, C and D, and Figure 3, C and D). Intriguingly, corresponding mRNA levels for these proteins did not show similar reductions (Figure 5B). Since podocyte density remained largely unchanged in MN (Figure 1F and Figure 3F), these findings suggest that reduced protein levels may not be driven by podocyte loss or transcriptional downregulation, but rather through posttranscriptional mechanisms, likely involving enhanced protein turnover and degradation.

To explore this possibility, we examined the involvement of proteolytic and protein degradation systems. The proteolytic pathways, especially the ubiquitin-proteasome system (UPS), were highly enriched in both transcriptomic and proteomic data (Supplemental Figure 7). The UPS and the autophagy-lysosomal system are major pathways maintaining cellular homeostasis, including in podocytes (34). Moreover, the superfamily of metalloproteinases, including ADAMs (a disintegrin and metalloproteinases), ADAMTS (a disintegrin and metalloproteinase with thrombospondin motifs), and MMPs (matrix metalloproteinases), catalyze extracellular matrix remodeling and pathological tissue changes (35). We found marked upregulation of various proteolysis-associated gene products in both proteomic and transcriptomic datasets (Figure 5, C and D). In particular, ADAM10, 12, 15, and 17, previously implicated in protein shedding during glomerulonephritis and fibrotic kidney disease (36–39), were markedly increased. Notably, ADAM10 has recently been shown to cleave both THSD7A and PLA2R1 (40). Additionally, multiple genes linked to the UPS were elevated in MN.

To validate these findings and to define their spatial localization, we performed immunofluorescence staining for ADAM15, the most upregulated metalloproteinase in MN mice (Figure 5C). The results confirmed a significant increase of ADAM15 within the glomeruli of MN mice compared with controls, with a prominent enrichment in podocytes (Figure 5E).

Together, these findings point to a potential role for specific protein degradation systems in the pathogenesis of MN, possibly through the direct or indirect promotion of critical SD protein loss, such as nephrin and NEPH1.

### Complement C3 deficiency attenuates proteinuria but not SD protein loss in MN.

**Figure 6:**
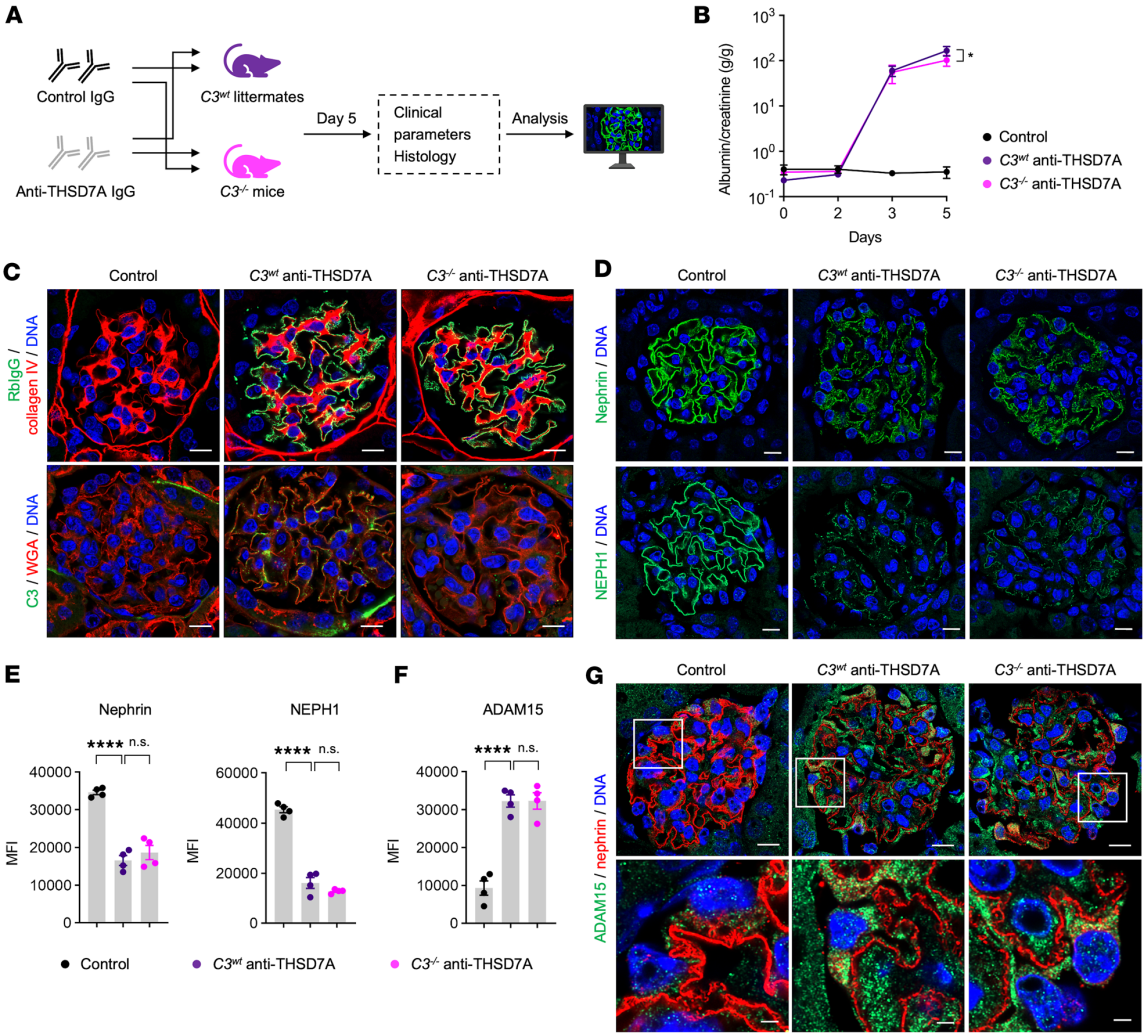
Complement C3 deficiency attenuates proteinuria but not slit diaphragm protein loss in MN. (**A**) Experimental workflow for *C3^wt^* controls and *C3^–/–^* mice injected with control and anti-THSD7A IgG. (**B**) Albumin-to-creatinine ratio (g/g) over time. *P < 0.05 (*C3^wt^* anti-THSD7A IgG vs. *C3^–/–^* anti-THSD7A IgG) (2-way ANOVA with Bonferroni’s multiple-comparison test). Data shown as mean ± SEM, *n* = 4 per group. (**C**) Representative immunofluorescence stainings for rabbit IgG and collagen IV (upper panel), and C3 and WGA (lower panel) in control group and anti-THSD7A IgG–treated *C3^wt^* and *C3^–/–^* groups. Scale bars: 10 μm. (**D** and **E**) Immunofluorescence stainings of nephrin (**D**, upper panel) and NEPH1 (**D**, lower panel), and quantification (MFI) (**E**). Scale bar: 10 μm. ****P < 0.0001 (1-way ANOVA with Tukey’s multiple-comparison test). Data shown as mean ± SEM, *n* = 4 per group. (**F** and **G**) Immunofluorescence stainings of ADAM15 (**G**) and quantification (MFI) (**F**). Scale bars: 10 μm (upper zoom-out panels); 2 μm (lower zoom-in panels). ****P < 0.0001 (1-way ANOVA with Tukey’s multiple-comparison test). Data shown as mean ± SEM, *n* = 4 per group; n.s., not significant.

To further dissect whether the observed effects in regard to SD protein loss and activation of proteolytic pathways depend on activation of the complement system, we next induced the MN model in genetically C3-deficient mice (*C3^–/–^* mice) and their *C3^wt^* littermates (Figure 6A). We used a control group consisting of both *C3^–/–^* and *C3^wt^* littermates, which received control rabbit IgG. After the injection of anti-THSD7A IgG, both *C3^wt^* and *C3^–/–^* mice developed significant albuminuria compared with the control group, and albuminuria was reduced by 38% in *C3^–/–^* MN mice at day 5 (mean albumin-to-creatinine ratio at day 5: 166.8 ± 39.8 vs. 103.4 ± 27.7 g/g in *C3^wt^* and *C3^–/–^* mice, respectively, Figure 6B). Anti-THSD7A–injected animals exhibited marked deposition of rabbit IgG along the glomerular filtration barrier (Figure 6C). As expected, C3 deposition was detected only in *C3^wt^* MN mice (Figure 6C).

Notably, the SD proteins nephrin and NEPH1 (Figure 6D) were significantly decreased in both MN groups, whereas no significant difference was identified between *C3^wt^* and *C3^–/–^* mice (Figure 6E). Furthermore, the metalloproteinase ADAM15 was equally upregulated in *C3^wt^* and *C3^–/–^* MN mice MN mice (Figure 6, F and G). Together, these experiments indicate that the loss of SD proteins and proteolytic activation are not a consequence of complement-mediated injury, but represent distinct, complement-independent pathomechanisms.

### THSD7A interacts with integrins in glomeruli.

**Figure 7:**
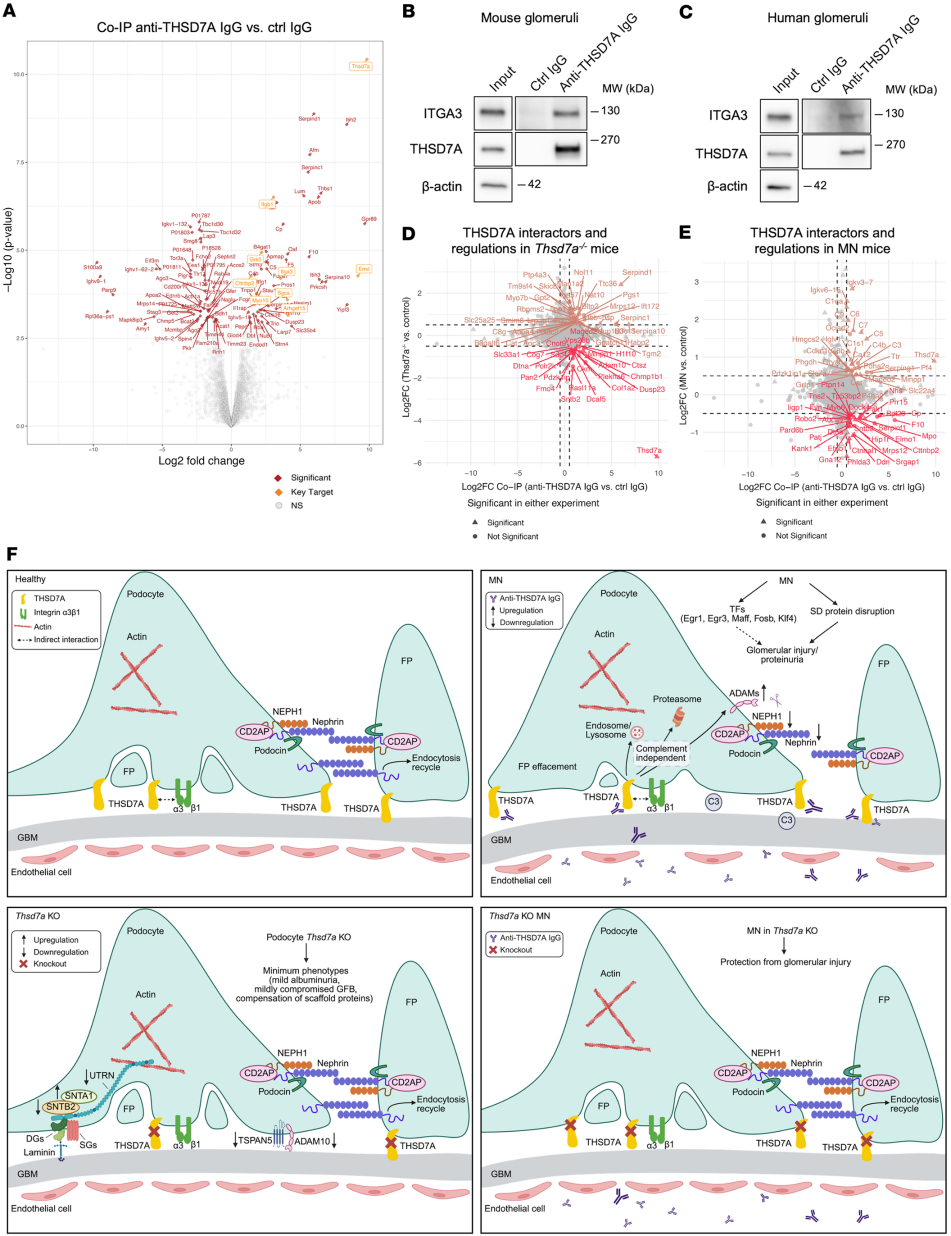
Integration of the THSD7A interactome with glomerular proteome dynamics and schematic model in MN. (**A**) Volcano plot showing the THSD7A interactome generated by co-IP/mass spectrometry from mouse glomeruli. –log_10_ *P* value is plotted against the log_2_ FC (THSD7A vs. control co-IP). Significant interactors are shown in red (limma moderated 2-tailed *t* test, Benjamini-Hochberg–adjusted *P* < 0.05, |log_2_FC| > 0.5); THSD7A and key interactors are highlighted in orange. (**B** and **C**) Co-IP validating integrin a3 (ITGA3) as a THSD7A interactor in mouse glomeruli (**B**) and human glomeruli (**C**) using anti-THSD7A IgG purified from patients with MN, and IgG from a healthy individual as a control. (**D** and **E**) Scatter plots comparing log_2_FC of the THSD7A co-IP/mass spectrometry interactome (*x* axis) versus log_2_FC of proteome (*y* axis) in *Thsd7a^–/–^* mice versus control mice (**D**), and MN versus control mice (**E**). Orange dots indicate proteins upregulated in both datasets, and red dots represent proteins upregulated in interactome while downregulated in proteome. Proteins significant in either dataset are shown as triangles, all others as circles. (**F**) Schematic model of glomerular dynamics in *Thsd7a^–/–^* and MN mice. Upper left: In healthy glomerulus, THSD7A localizes in podocyte foot processes (FPs) near slit diaphragm (SD) and indirectly associates with integrin α3β1. SD proteins maintain filtration barrier integrity. Upper right: in MN, SD disruption occurs without transcriptomic downregulation. Dysregulated protein degradation (ubiquitin-proteasome system, lysosomal proteolysis, proteases) and transcriptional reprogramming contribute to glomerular injury and proteinuria. Lower left: podocyte *Thsd7a^–/–^* mice show mild phenotypes featuring albuminuria and compromised glomerular filtration barrier, with compensation of scaffold proteins (SNTA1, SNTB2, UTRN) and transmembrane proteins (TSPAN5-ADAM10 complex). Lower right: *Thsd7a^–/–^* prevents anti-THSD7A IgG–induced MN phenotypes. GBM, glomerular basement membrane; TFs, transcription factors; C3, complement C3; DGs, dystroglycans; SGs, sarcoglycans. Created in BioRender.

In order to identify interactors of THSD7A in mouse and human glomeruli, we conducted co-IP experiments using mouse glomerular lysates and anti-THS7DA antibodies purified from patients with MN, followed by label-free quantitative data–independent mass spectrometry analysis. Among the 3,850 quantified proteins, 55 were significantly enriched in the anti-THSD7A co-IP group. Besides THSD7A itself, which was enriched approximately 1,000-fold, notable interactors included integrin α3 (ITGA3), integrin β1 (ITGB1), and podocyte-specific proteins such as the actin-based motor protein MYO10 and cortactin binding protein 2 (CTTNBP2) (Figure 7A). Additional potential binding partners included the nuclear membrane protein emerin, known for its actin-binding properties (41); α-sarcoglycan (SGCA), a component of the dystrophin-glycoprotein complex essential for linking the actin cytoskeleton to the extracellular matrix (42); Rho guanine nucleotide exchange factor 15 (ARHGEF15), which regulates RHO-family GTPases including RHOA, CDC42, and RAC (43, 44); and the G protein–coupled receptor kinase GRK5, a serine/tyrosine kinase involved in GPCR regulation (45). Proteins uniquely detected in the anti-THSD7A IgG co-IP group, but absent in the control IgG group, included additional putative interactors such as PODXL, CD81, and ROBO4 (Supplemental Figure 8A).

These findings align with previous research implicating THSD7A in cytoskeletal regulation, focal adhesion, and membrane dynamics across various cell types, including podocytes (5, 6). Our results suggest that THSD7A plays a key role in the structural membrane organization of podocytes through interactions with these partners. In particular, integrin α3 and β1 usually form the heterodimeric integrin α3β1 receptor, which is critical for podocyte adhesion to the GBM (46). We further validated the specific interaction between THSD7A and ITGA3 in both mouse and human glomeruli by immunoblotting (Figure 7, B and C). Moreover, we tested whether THSD7A and ITGA3 interact directly by performing IP using recombinant protein instead of glomerular extracts. We found that THSD7A does not bind ITGA3 directly (Supplemental Figure 8B), suggesting that these 2 proteins are associated within a larger protein complex.

Integrating the THSD7A interactome with glomerular proteomes from podocyte-specific *Thsd7a^–/–^* mice and MN mice revealed disease-relevant regulatory patterns. Notably, proteins such as SNTB2 and ADAM10, which were downregulated in glomeruli of *Thsd7a^–/–^* mice, were enriched in THSD7A co-IP, suggesting functional and/or physical interactions with THSD7A (Figure 7D). Comparative analysis between the THSD7A interactome and MN proteome uncovered dysregulated interactors implicated in MN pathogenesis (Figure 7E), including proteins involved in RHO GTPase signaling (DOCK4, PAK1, PARD6B, MYO6, NHS, SRGAP1), ephrin signaling (EFNB1, PAK1, FYN), and axon guidance (ROBO2, EFNB1, PAK1, PARD6B, FYN, SRGAP1). Interestingly, we observed an enrichment of CTTNB2 in co-IP and a reduced expression of CTTNBP2 in MN. Given its role as a microtubule-stabilizing THSD7A interactor, our findings suggest its involvement in anti-THSD7A IgG–mediated cytoskeletal disruption.

Collectively, we validated THSD7A interactors in native mouse and human glomeruli and established their potential contributions to MN pathogenesis via dysregulation of cytoskeletal organization, focal adhesion dynamics, and membrane stability.

## Discussion

THSD7A is a critical autoantigen in patients with MN (2); however, its intrinsic role in podocyte biology has remained incompletely understood. Here, we show that THSD7A critically contributes to MN pathogenesis, while podocyte-specific loss of THSD7A causes only mild phenotypic alterations.

To uncover mechanisms of anti-THSD7A antibody–mediated injury, we applied multiomics, including transcriptomics and proteomics, in a passive transfer MN model. Bulk RNA-Seq at peak injury (day 5 after injection) revealed prominent changes in podocyte-specific genes and disruptions in gene products regulating podocyte adhesion and cytoskeleton dynamics. Key pathways implicated included extracellular matrix–receptor interactions, RHO GTPase signaling, transcription factor activity, and cell cycle and DNA damage/repair, which characterize antibody-mediated podocyte injury in MN (Figure 7F).

Integration of transcriptomic and proteomic data revealed stable mRNA levels but markedly reduced protein abundance of key SD components such as nephrin, NEPH1, podocin, and ephrin B1. This critical finding led us to hypothesize that loss of SD proteins may be driven by posttranscriptional mechanisms involving altered protein turnover and degradation, rather than transcriptional downregulation. In line with this hypothesis, our multiomics analysis pointed toward increased proteolytic activity in MN, including regulation of UPS activity, upregulation of lysosomal proteases (cathepsins S/Q), cytosolic calpain 5/S1, and ADAM proteases (ADAM10/15/17), all previously linked to glomerular injury (Figure 7F) (37, 47–49). The pathogenic roles of these systems are well described. For instance, although recombinant cathepsin S alone does not directly induce podocyte injury in vitro, its inhibition attenuates SD protein loss and albuminuria in diabetic mice, consistent with clinical associations with chronic kidney disease progression (47, 48). Similarly, ADAM17 mediates podocyte injury through ectodomain shedding of adhesion molecules, and its KO prevents loss of nephrin and podocin in diabetic kidney disease models (36), aligning with our findings in THSD7A-associated MN. ADAM15, the most upregulated ADAM protease in our data, is known to degrade collagen IV and remodel mesangial matrix (38). Additionally, activation of UPS may also represent another potential mechanism for SD protein loss, as podocin and NEPH1 are known to undergo ubiquitination, and proteasomal inhibition affects nephrin membrane levels by disrupting endocytic turnover (50, 51).

This degradation imbalance observed in MN may be attributed to the anti-THSD7A IgG deposits interfering with the physiological recycling-degradation equilibrium in podocytes. Normally, nephrin and other SD proteins are tightly regulated via endocytosis and recycling to maintain proper surface expression levels and SD integrity (52). Extensive IgG binding at the podocyte membrane may skew this process toward excessive degradation via lysosomal, ADAM, or UPS pathways, ultimately compromising SD integrity. Therapeutic targeting of overactive proteases or UPS components could restore this balance, offering a strategy to mitigate proteinuria and podocyte loss in MN.

Another finding from our integrated multiomics analysis was the strong enrichment of DNA replication and cell cycle pathways. This was associated with upregulation of DNA damage response, cell cycle arrest, and senescence markers, suggesting a maladaptive pathway driving initial podocyte or glomerular dysfunction.

The pathogenesis of MN has long been centered on complement-mediated podocyte injury. Our study now supports a dual-pathomechanism in THSD7A-associated MN. Although the established complement-dependent pathway contributes to proteinuria, as shown by its amelioration in this and other C3-KO or C3-inhibited MN models (11, 12, 53), our findings reveal a critical and parallel complement-independent pathway. Key evidence comes from our *C3^–/–^* mice, which showed profound SD protein loss and activation of proteolytic systems. These results indicate that the identified injury mechanisms are not merely secondary to complement activation. Instead, they likely represent a direct injury pathway triggered by anti-THSD7A antibodies, possibly through disruption of normal podocyte function (53).

In contrast to the profound injury induced by anti-THSD7A antibodies, podocyte-specific THSD7A deficiency caused only mild changes: modest albuminuria and foot process widening without significant podocyte loss. Proteomic profiling confirmed stable expression of key SD proteins in the absence of THSD7A, indicating that in mice, THSD7A is not as critical as nephrin, podocin, or NEPH1 in maintaining filtration barrier function under physiological conditions (16). This divergence suggests that THSD7A may not directly govern filtration barrier stability under physiological conditions and instead may be responsible for subtle regulation of podocyte structure and function.

Interestingly, several podocyte-specific scaffold proteins, including syntrophins, utrophin, and TSPAN5, were altered in *Thsd7a^–/–^* mice. SNTA1, the major syntrophin isoform, and SNTB2 directly bind F-actin and regulate cytoskeletal dynamics (54), while utrophin anchors the cytoskeleton to the GBM (55, 56). The upregulation of syntrophins and dysregulation of utrophin in *Thsd7a^–/–^* mice likely reflect a compensatory remodeling of scaffold networks to stabilize SD architecture in the absence of THSD7A, which may explain the relatively mild phenotype observed (Figure 7F).

We hypothesize that THSD7A functions as a critical adaptor in podocytes via protein interactions. The most compelling evidence emerged from co-IP experiments demonstrating a robust association between THSD7A and integrin α3 in both mouse and human glomeruli (Figure 7F). Our data support an indirect interaction between THSD7A and integrin α3. This indirect linkage is compatible with super-resolution imaging that places the 2 proteins in adjacent but spatially separated membrane domains of podocytes (5). In further support of a multiprotein complex, we detected co-association of known integrin α3β1 partners, including tetraspanins CD81 and CD151 — a known mediator of podocyte-GBM adhesion (57) (log_2_ fold-change = 3.44, *P* = 0.03 in co-IP). Given that integrin α3β1 is the principal laminin receptor essential for podocyte adhesion and structural integrity (58), and that THSD7A overexpression enhances podocyte adhesion and reduces migration (5), we propose a model in which integrin α3β1 acts as the bridge linking a THSD7A-containing complex to the extracellular matrix. Moreover, the interactions of THSD7A with molecules such as sarcoglycan complex SGCA (linking F-actin cytoskeleton and extracellular matrix) (59), molecular motor MYO10 (localized to filopodia tips) (60), actin-binding and polymerization-promoting protein emerin (41), and RHO GTPase ARHGEF15 suggest THSD7A as a central hub coordinating extracellular matrix sensing and cytoskeletal remodeling in podocytes.

Taken together, our integrated analysis suggests that multiple processes, including SD disruption and protein degradation, contribute to MN pathogenesis, while loss of THSD7A alone induces only mild podocyte alterations. Notably, PLA2R1, another major MN antigen, is also dispensable in rodent podocytes (61), suggesting both antigens may not be critical for homeostasis. This may explain the chronic and slowly progressive nature of MN, which usually manifests after prolonged autoantibody exposure (62). This stands in contrast to, for example, the rapid onset of anti-nephrin–associated podocytopathy, where anti-nephrin autoantibodies directly disrupt essential SD function and signaling (63, 64). Silencing or degrading THSD7A and PLA2R1 with antisense oligonucleotides, siRNAs, adeno-associated viral vectors, or PROteolysis TArgeting Chimeras (PROTACs) could represent potential therapeutic approaches to reduce antigen availability and prevent antibody-mediated injury, though challenges in delivery and safety still remain (65–68).

A limitation of our study is the use of bulk RNA-Seq and proteomics from whole glomeruli, which does not allow cell type–specific resolution. Future single-cell or spatial omics approaches will provide deeper insights into MN pathomechanisms. In addition, the critical involvement of protein degradation pathways suggested by our multiomics analysis requires further functional validation in future studies. It is also important to note that the genetic C3 deficiency in the mice used for this study can lead to developmental adaptations of the immune system (69).

In summary, we provide comprehensive transcriptomic and proteomic profiles of antibody-induced glomerular injury in MN. Our data, particularly a notable posttranscriptional discrepancy for key SD proteins, led us to identify and validate the activation of proteolytic degradation as a significant pathological component. A key finding is the delineation of a complement-independent pathway, which drives SD damage and proteolytic activation, alongside the classical complement-dependent injury pathway. We demonstrate THSD7A as a podocyte scaffold protein that associates with integrin α3, likely within a larger molecular complex, coordinating adhesion and cytoskeletal stability. Although dispensable for normal podocyte function, THSD7A deficiency protects from anti-THSD7A antibody–induced injury (Figure 7F). These insights lay a foundation for an improved understanding of MN and propose targeted antigen downregulation and proteostasis modulation as potential therapeutic strategies.

## Methods

### Sex as a biological variable.

For the characterization of *Thsd7a^–/–^* mice, both male and female mice were examined. Given that no significant differences were found between male and female mice, results are reported for both sexes together. In the MN model, only male mice were investigated because MN has a strong dominance for males, and male mice show less variability in phenotype. It is unknown whether the findings in these experiments are relevant for female mice as well.

### Animal experiments.

Podocyte-specific *Thsd7a^–/–^* mice were established using a KO-first strategy (70) as detailed in the Supplemental Appendix, and *C3^–/–^* mice were generated as previously described (12). Experimental MN was induced as previously described (15).

### Histological analyses.

Immunofluorescence staining, PAS staining, and electron microscopic analyses were performed as described in the Supplemental Appendix.

### Co-IP.

For co-IP, glomerular lysates were incubated with either anti-THSD7A IgG purified from patients with THSD7A-associated MN or the same amounts of human control IgG (Aviva Sysbio, OASB01433) followed by addition of protein G agarose beads (GenScript) overnight, as detailed in the Supplemental Appendix.

### qRT-PCR and bulk RNA-Seq.

Total RNA was extracted from mouse glomeruli using RNeasy Mini kit (QIAGEN, 74104), and qRT-PCR was performed as detailed in the Supplemental Appendix. Bulk RNA-Seq libraries were prepared from 500 ng RNA (Novogene NGS RNA Library Prep Set, PT042) and sequenced (150 bp paired-end, NovaSeq 6000) by Novogene. After quality assessment and trimming, paired-end clean reads were aligned to a mouse reference genome (GRCm39, Ensembl 104) and quantified with Kallisto (version 0.46.2) (71). Differential expression analysis was performed using DESeq2 (version 1.38.3) (72) in R (version 4.2.0) (73). Genes with a Benjamini-Hochberg–adjusted *P* value less than 0.05 and an absolute log_2_ fold-change greater than 0.5 were designated as differentially expressed. Heatmaps were plotted using R package Complexheatmap (version 2.15.4) (74). Protein-protein interaction networks were generated using STRINGdb (22). Enrichr (75) and R package fgsea (76) were used for pathway enrichment.

### Proteomics.

Isolated glomeruli for proteomics were prepared as described in the Supplemental Appendix. Raw data were queried using Biognosys Spectronaut 19.0.240606.62635 (Huggins) and the canonical reference proteome for mice from UniProt (UP000000589_10090, 21,949 entries, March 2023) at standard settings. Enzyme specificity was set to trypsin/P, cysteine carbamidomethylation was set as a fixed modification (+57.021464), and methionine oxidation (+15.994914) and protein N-terminal acetylation (+42.010565) were set as variable modifications. Identification was performed with the “directDIA+ (Deep)” workflow, quantification was filtered by “Identified (Qvalue)” for the precursors, and no imputation was carried out. The Spectronaut outputs (normalized log_2_ quantities) were further processed using the DEP2 package (77) in R and RStudio, which internally use limma (78) for comparison testing. We applied a threshold for significance based on a Benjamini-Hochberg–adjusted *P* value less than 0.05 and an absolute log_2_ fold-change greater than 0.5. Gene set enrichment analysis was performed with R packages fgsea and ClusterProfiler (79) as implemented in the DEP2 package.

### Statistics.

Animals were assigned to treatment groups randomly and age-matched between conditions where necessary. Statistical analysis was conducted using GraphPad Prism. Data are shown as mean ± SEM. Differences between 2 groups were analyzed using an unpaired 2-tailed *t* test. Differences between 3 groups or more were calculated using 1-way ANOVA with a post hoc Tukey’s multiple-comparison test. Comparisons with multiple variables were analyzed using a 2-way ANOVA with Bonferroni’s multiple-comparison test. In cases of missing values, differences were analyzed using a mixed-effects analysis with Bonferroni’s multiple-comparison test. Details on experimental replicates and animal numbers are demonstrated in figure legends. A *P* value less than 0.05 was considered significant.

### Study approval.

Animal experiments were approved by the Veterinarian Agency of Hamburg and the local animal care committee (registration N116/2020). The use of human serum for IP studies was approved by the local ethics committee of the Chamber of Physicians in Hamburg. Patients gave written and informed consent.

### Data availability.

The differential expression analyses of mass spectrometry data are available in Supplemental Tables 1–3. Raw mass spectrometry proteomics data and Spectronaut outputs have been deposited to the ProteomeXchange Consortium via the PRIDE partner repository (https://www.ebi.ac.uk/pride) with the accession numbers PXD065322, PXD065323, and PXD065344. The bulk RNA-Seq data have been deposited in NCBI’s Gene Expression Omnibus (GEO GSE300256). The differential expression analysis of transcriptomics data is provided in Supplemental Table 4. All data used to create the graphs in the manuscript are available in the Supporting Data Values file.

## Author contributions

GZ, MMR, and NMT jointly supervised the study. MH, ML, SD, FEH, FD, AMB, NS, LS, OK, SB, and GZ performed experiments. FG, UP, and TBH provided expertise and reagents. MH, ML, and MMR analyzed the data. MH and ML wrote the first draft of the manuscript. All authors revised and approved the last version of the manuscript. MH and ML contributed equally as co–first authors. MH conducted the mouse work and transcriptomics analysis, and ML performed the proteomics analysis. MH is listed first for her additional role in coordinating the integration of the datasets and revisions. GZ, MMR, and NMT made equal contributions to this manuscript as last authors.

## Conflict of interest

FEH reports honoraria from Novartis. TBH reports consulting fees from Boehringer Ingelheim, Novartis, Alexion, Pfizer, Retrophin-Travere, and Fresenius Medical Care. MMR reports research funding from Novo Nordisk to Aarhus University unrelated to this topic. NMT reports a patent in relation to the measurement of anti-THSD7A antibodies (EP2015/066881); consultancy fees from Merida Bioscience; and honoraria from CSL Vifor, StreamedUp, Novartis, and AstraZeneca.

## Funding support

- Deutsche Forschungsgemeinschaft (DFG) as part of the collaborative research center TRR 422 PodoSigN (to FG, SB, TBH, MMR, and NMT).
- DFG as part of the collaborative research center 1192 (to FEH, FG, UP, TBH, MMR, and NMT).
- Else Kröner Fresenius Foundation (to FEH).
- DFG (GR 3933/1-2, to FG).
- DFG (RI 2811/1-1, RI 2811/2-1 and FOR2743, to MMR).
- Young Investigator Award from the Novo Nordisk Foundation (NNF19OC0056043, to MMR).
- Carlsberg Young Investigator fellowship (to MMR).
- Aarhus University Research Foundation (to MMR).
- NMT (Emmy Noether Program, to NMT).
- European Research Council (AUTO-TARGET, to NMT).
- Else Kröner Fresenius Foundation (Clinician Scientist Professorship, to NMT).
- European Union.

## Supplementary Material

Supplemental data

Supporting data values

## Version 1 02/26/2026

In-Press Preview

## Version 2 04/22/2026

Electronic publication
