# Supplementary material for: Functional characterization of podocyte-expressed THSD7A in experimental membranous nephropathy
Source: JCI Insight. 2026 Feb 26;11(8):e198363. doi: 10.1172/jci.insight.198363 (PMC13135397; doi:10.1172/jci.insight.198363)
Supplement: Supplemental data [file jciinsight-11-198363-s106.pdf]

Supplementary Appendix

for

Functional characterization of podocyte-expressed THSD7A in experimental membranous nephropathy

Ming Huang*, Moritz Lassé*, Silke Dehde, Felicitas E. Hengel, Fatih Demir, Anja Billing, Ning Song, Larissa Seifert, Oliver Kretz, Florian Grahammer, Ulf Panzer, Sebastian Brähler, Tobias B. Huber, Gunther Zahner#, Markus M. Rinschen#, and Nicola M. Tomas#

Supplementary Methods

Animal experiments

All mice were bred in the animal facility of the University Medical Center Hamburg-Eppendorf. Animals were housed in a specific-pathogen-free facility at temperatures of 21~24 °C on a 12 h day/night cycle and had free access to standard laboratory chow and water. Podocyte specific *Thsd7a* knockout (KO) mice were established using 'knockout-first' strategy (1). *Thsd7a* knockout first mice (*Thsd7a*^{tm1a} mice) containing lacZ trapping cassette and a floxed promoter-driven neo cassette in intron of *Thsd7a* were obtained from the Mutant Mouse Resource & Research Centers, MMRRC, stock no. 063558-UCD). *Thsd7a*^{tm1a} mice were firstly bred with Flp recombinase mice, generating *Thsd7a*^{tm1c} (Cre^{-}/*Thsd7a*$^{fl/fl}$ mice) mice with restored *Thsd7a* activity. *Thsd7a*^{tm1c} mice were then crossed with podocin-Cre recombinase mice to generate podocyte specific *Thsd7a* knockout mice (Cre^{+}/*Thsd7a*$^{fl/fl}$). The littermates without podocin-Cre (Cre^{-}/*Thsd7a*$^{fl/fl}$ mice) were used as control. In parallel, mice with podocin-Cre recombinase expression (Cre^{+}/*Thsd7a*$^{+/+}$) were also included as control in long-term basic characterization experiments. *Thsd7a* podocyte knockout mice were backcrossed to the BALB/c background for ten generations. Genotyping was performed using the protocol and primers suggested by the provider. We monitored the basic parameters of knockout mice and control littermates as long as 12 months.

To generate C3 deficient (*C3*$^{-/-}$) mice on the BALB/c background, the B6;129S4-*C3*^{tmCrr}/J strain (stock no. 003641, The Jackson Laboratory) was backcrossed for ten generations onto wild-type BALB/c mice, resulting in a C.Cg.*C3*^{tm1Crr}/J mouse line. For experiments, mice with a heterozygous C3 status were bred to generate homozygous *C3*$^{-/-}$ mice as well as *C3*^{wt} littermate controls. Genotyping was conducted according to the protocol and primers suggested by the provider. For the induction of THSD7A-associated membranous nephropathy (MN), 14 to 15-week-old male *Thsd7a* podocyte knockout and their wildtype littermate mice, or *C3*$^{-/-}$and *C3*^{wt} littermates were injected intraperitoneally with 2.0 mg of either rabbit control IgG or rabbit anti-THSD7A IgG. Mice used for bulk RNA sequencing were euthanized after 1 and 5 days, and mice injected with control IgG at both time points were combined into a single control group for analysis. Mice used for proteomic characterization were euthanized after 5 days of injection. Urine samples were collected using metabolic cages and body weight was recorded before urine collection. Urinary albumin content was measured using a commercially available ELISA system (Bethyl Laboratories) according to the manufacturer's instructions. Urinary creatinine levels were determined with the Jaffé reaction (Creatinine Jaffé Kinetisch, WAK-Chemie). The urine albumin concentrations were standardized against the urinary creatinine concentrations to obtain albumin to creatinine ratios.

Immunofluorescence staining

Paraffin sections (3 µm) of mouse kidneys were deparaffinized and rehydrated. Antigen retrieval was performed by boiling in Dako Target Retrieval buffer (Dako, Carpinteria) at pH 9 or pH 6 for 30 minutes in a steamer at 98 °C, or by digestion with protease XXIV (5 µg/mL; Sigma-Aldrich) for 15min at 37 °C. Unspecific binding was blocked with 5% horse serum (Vector Laboratories) and 0.05% Triton X-100 (Sigma-Aldrich) in PBS for 30 minutes at room temperature. Primary antibodies for murine sections (THSD7A [rabbit, 1:200; Sigma-Aldrich HPA000923 or mouse, 20 µl/ml; in-house generated anti-mouse THSD7A d15 domain monoclonal antibody conjugated with FITC fluorochrome (2)], nephrin [guinea pig, 1:200; Progen GP-N2], DACH1 [rabbit, 1:100; Sigma-Aldrich HPA012672], synaptopodin [guinea pig, 1:200; Synaptic Systems 163004], NEPH1 [guinea pig, 1:200; in-house generated (3)], C3 [FITC goat anti-C3, 1:100; Cappel 55500], ADAM15 [goat, 1:50; R&D AF945], collagen IV [goat, 1:400, SouthernBiotech 1340-01], rabbit

IgG [Cy5-RbIgG H+L, 1:100; Jackson ImmunoResearch Laboratories 111-175-144]) and biotinylated LTL (1:200; Vector Laboratories B-1325-2) were diluted in blocking buffer and incubated at 4 °C overnight. Binding of primary antibodies was visualized by subsequent incubation of sections with affinity purified, fluorochrome-conjugated secondary antibodies (1:200; Jackson ImmunoResearch Laboratories, AF488 anti-goat IgG [705-545-147], AF488 anti-rabbit IgG [711-545-152], Cy3 anti-rabbit IgG [711-165-152], Cy2 anti-guinea pig IgG [706-225-148], Cy3 anti-guinea pig IgG [706-165-148]), Streptavidin-Alexa Fluor™ 647 Conjugate (1:100; Thermo Scientific S21374), WGA-rhodamin (1:400; Vector-Laboratories RL-1022-5), Hoechst 33342 (1:1000; Sigma-Aldrich AMBH9A260690) or DRAQ5 (1:1000; Cell Signaling 4084) for 30 minutes at room temperature in blocking buffer. Sections were then mounted with Fluoromount-G (Invitrogen). Stainings were evaluated using the inverted laser confocal microscope LSM 800 from Zeiss. Representative images were obtained using the Airyscan of the ZEN 2.6 (blue edition) software with 63× or 40× oil immersion objectives. Nephrin, NEPH1 and synaptopodin, ADAM15 intensity was quantified using ImageJ. For each mouse, 10 glomeruli were manually outlined, and the mean fluorescence intensity (MFI) was measured for each marker using a consistent threshold setting across all images. For quantification of podocyte density, DACH1 positive nuclei per glomeruli were quantified. In total, 15 glomeruli per mouse were used for quantification, and the ratio of podocyte number to glomeruli area was assessed using Image J. This ratio was termed “podocyte density”. MFI or podocyte density from each mouse were averaged and plotted in the corresponding figures.

Periodic acid-Schiff (PAS) staining

Paraffin sections (1.5 µm) of mouse kidneys were deparaffinized and rehydrated. Sections were incubated in 1% periodic acid (HIO_4) for 15 minutes to oxidize diols to aldehydes, followed by incubation of Schiff reagent (Sigma) for 40 minutes at room temperature. Counterstaining of nuclei was then performed with hemalaun and following dehydration sections were mounted with Eukitt (O. Kindler GmbH). The PAS-sections were evaluated with an Axioskop using the Axiovision software for light microscopy (Zeiss).

Electron microscopy

Electron microscopic analyses were performed on mouse renal cortex pieces that were fixed in 4% buffered paraformaldehyde. Tissue blocks were treated with 1% osmium in 0.1M sodium-cacodylate buffer, stained with 1% uranyl acetate and embedded in epoxy-resin (Serva). Ultrathin sections were cut using an Ultramicrotome (Reichert-Jung) and contrasted with uranyl acetate in methanol followed by lead citrate. Micrographs were generated with a transmission-electron microscope (JEM 1010, JEOL).

For quantification of podocyte foot process (FP) width, the glomerulus basement membrane (GBM) was traced and measured by Image J in each picture (4). The number of FPs along the GBM was counted manually. The arithmetic mean of FP width was calculated using this formula (4):

$$FPwidth = \frac{\Sigma GBM\ length}{\Sigma\ \text{foot process}} \cdot \frac{\pi}{4}$$

For each mouse, the mean width of FP was calculated for plotting and statistics.

Glomeruli isolation and tissue preparation

Mice were anesthetized under isoflurane, and both kidneys were harvested and perfused with Dynabeads (Thermo Scientific 14013) in HBSS through the renal artery. Glomeruli were isolated using either of two approaches, i) enzymatic

digestion or ii) mechanical isolation. In the enzymatic digestion method, perfused-kidneys were minced into small pieces using a scalpel blade, and transferred to a C-tube (130-093-237, Miltenyi Biotec) filled with 5 ml collagenase solution (1 mg/ml collagenase from *Clostridium histolyticum* [C9263, Sigma-Aldrich], 0.25% bovine serum albumin [BSA], 100 U/ml DNaseI in DMEM/F-12 medium [11320074, Gibco]). Kidney tissues were digested for 5 minutes at 37°C followed by homogenization using a gentleMACS^{TM} Dissociator (Miltenyi Biotec) with program "m.spleen 01_01". This procedure was repeated once. Alternatively, to avoid the collagenase digestion and potential cleavage of some proteins, mouse glomeruli used for co-immunoprecipitation were isolated mechanically using a Minilys system (Bertin). Briefly, Dynabeads-perfused-kidneys were minced with a scalpel blade and dissociated in a Precellys® Tube (prefilled with 2.8 mm ceramic beads, CK28/ 2ml, VWR) filled with HBSS using a Minilys machine at 5000 rpm for 15 seconds. The kidney suspension was gently pressed through a 300 µm and a 100 µm cell strainer and washed with HBSS. After centrifugation at 300 g for 5 minutes, the pellet was resuspended and glomeruli containing Dynabeads were collected by a magnetic particle concentrator (Invitrogen). Glomeruli were washed multiple times with 0.05% BSA-HBSS solution and counted using a cell chamber under microscope. The glomeruli pellet was snap frozen and stored at -80 °C until further use.

The use of de-identified human kidney tissue was deemed exempt from Institutional Review Board oversight, as no identifiable information was used, and materials were obtained from anonymized specimens. Human glomeruli isolation was performed according to previous descriptions with modifications (5, 6). Macroscopically healthy parts of kidneys from patients who underwent nephrectomy were used for the preparation of glomeruli. Minced kidney cortex was sieved through pre-wetted 150 µm strainers, followed by extensively washing with PBS-0.05% human albumin (CSL Behring). The flow-through was cleaned from small tissue components using a 90 µm strainer. Glomeruli were rinsed off the strainers, quantified by manual counting and pelleted at 1200 g for 15 minutes at 4 °C. Glomerular cell pellets were snap frozen and stored at -80 °C until further use.

Mouse tissues (kidney, heart, lung, liver, spleen, brain, skin, muscle, testis, intestine, and colon) were harvested and quickly frozen in liquid nitrogen following mouse anesthesia and cervical dislocation. Tissues were homogenized in RIPA buffer (50 mM Tris pH 7.4, 150 mM NaCl, 1% Nonidet P-40, 0.5% Sodium deoxycholate, 0.1% SDS) containing protease inhibitor (Inhibitor Cocktail plus, Carl Roth GmbH) and phosphatase inhibitors (8 mM NaF, 4 mM Na_3VO_4) using dounce homogenizers with 60 repetitions (Wheaton), followed by shearing with a 27-G needle. Lysates were centrifuged at 20,000 g for 10 minutes at 4 °C. Protein concentration was measured using DC Protein Assay according to the manufacturer's instruction (Bio-Rad) and stored in -80 °C for immunoblot analysis.

Immunoblotting

For immunoblot analysis, mouse glomeruli were lysed with Triton lysis buffer (20 mM Tris pH 7.4, 150 mM NaCl, 1% Triton X-100) supplemented with protease inhibitor (Carl Roth) and phosphatase inhibitors (8 mM NaF, 4 mM Na_3VO_4). After centrifugation at 20,000 g for 10 minutes, supernatant of lysates was mixed with 5x Laemmli buffer (1.5 M Tris-HCl, pH 6.8, 50% glycerol, 10% SDS, 1% bromophenol blue with or without 50 mM of DTT), heated at 95 °C for 10 minutes, and separated by SDS-PAGE using precast gradient gels (4-15% TGX, BioRad). Subsequently, the samples were transferred to methanol-activated PVDF membranes (EMD Millipore) under semi-dry conditions. Membranes were blocked with 5% dry milk in TBS-Tween 0.05% (TBS-T) at room temperature for 2 h followed by incubation with the primary antibodies at 4°C overnight. The following primary antibodies were used: THSD7A (rabbit, 1:1500; Sigma-Aldrich HPA000923), nephrin (guinea pig, 1:1500; Progen GP-N2), NEPH1 (guinea pig, 1:1000; in-house generated),

serum from patients with THSD7A-associated MN (1:100), integrin α3 (mouse, 1:1000; proteintech 66070-1-Ig), β-actin (mouse, 1:10,000; Sigma-Aldrich A2228), α-tubulin (mouse, 1:2000; Sigma-Aldrich T5168). Membranes were then washed in TBS-T and incubated with an appropriate HRP-conjugated secondary antibody in block solution (anti-mIgG, 1:10,000, Jackson ImmunoResearch Laboratories 715-035-151; anti-hIgG4, 1:20,000, Southern Biotech 9200-05; anti-rabbit IgG, 1:10,000, Jackson ImmunoResearch Laboratories 711-035-152; anti-guinea pig IgG, 1:10,000, Jackson ImmunoResearch Laboratories 706-035-148) for 1 h at room temperature. Membranes were developed with chemiluminescent substrate (SuperSignal West Pico; Thermo Scientific) followed by incremental luminescence detection with an Amersham Imager 600 (GE). Relative intensity of protein expression was quantified by optical density using the Quantity One software (Bio-Rad).

Co-immunoprecipitation

The use of human serum for immunoprecipitation studies was conducted in accordance with federal state and institutional guidelines as well as the Declaration of Helsinki and was approved by the local ethics committee of the Chamber of Physicians in Hamburg. Patients gave written and informed consent.

For co-immunoprecipitation (co-IP), glomerular lysates were incubated with either anti-THSD7A IgG purified from serum of THSD7A-associated MN patients or same amounts of human control IgG (Aviva Sysbio) at 4°C for 2 h followed by addition of protein G agarose beads (GenScript) for incubation at 4°C overnight. The immunoprecipitates with protein G-beads were washed five times in wash buffer 1 (50 mM Tris pH 7.5, 150 mM NaCl, 0.3% Triton) and three times in wash buffer 2 (50 mM Tris pH 7.5, 150 mM NaCl). The immunoprecipitates were eluted with SP3 elution buffer (4% SDS, 0.1 M HEPES, pH 7.4, 5 mM EDTA) containing 1x Halt Protease and Phosphatase Inhibitor Cocktail (Thermo Scientific). For immunoblotting detection, the eluted immunoprecipitates were boiled in 5x Laemmli buffer (with 50 mM of DTT) at 95 °C for 10 minutes.

Pull-down assays

The construct encoding the extracellular domains of mouse THSD7A (mTHSD7A) with a FLAG tag was generated as previously described (7-9). Briefly, the construct was generated by PCR from a full-length, FLAG-tagged mTHSD7A cDNA construct (Origene) and cloned into the eukaryotic expression vector pCSE2.5 (provided by Thomas Schirrmann, Braunschweig, Germany). For production of mTHSD7A extracellular domain protein, human embryonic kidney cells (HEK)293-6E cells (kindly provided by Yves Durocher, Ottawa, Canada (10)) were transfected using a polyethylenimine (PEI; Polyscience) based method (9), and supernatants were collected for purification after 6 days of transfection. The protein was purified using anti-FLAG M2 affinity resin (Sigma-Aldrich, A2220) according to the manufacturer's instructions. Recombinant His-tagged mouse integrin α3β1 (mITGA3-His), derived from Chinese hamster ovary (CHO) cell line, was purchased from R&D (9374-A3).

For pull-down assays, 300 ng of recombinant mITGA3-His protein was immobilized on 15 µl settled HisPur Ni-NTA resin (Thermo Scientific), and incubated with or without 300 ng of mTHSD7A extracellular domain protein in binding buffer (PBS pH 7.4, 10 mM imidazole, 0.3% Triton X-100, protease inhibitor) at 4 °C overnight. 300 ng of mTHSD7A extracellular domain protein incubated with HisPur Ni-NTA resin alone served as another control group. On the following day, flow-through (FT) fractions containing unbound proteins were collected, and the resin-bound proteins were washed three times in wash buffer1 (PBS pH 7.4, 10 mM imidazole, 150 mM NaCl, 0.3% Triton) and twice in wash buffer 2 (PBS pH 7.4, 10 mM imidazole, 150 mM NaCl). Proteins bound to resin were eluted in elution buffer

(250 mM imidazole in PBS, pH 7.4) at room temperature for 10 min, and then boiled in 5x Laemmli buffer containing 50 mM DTT at 95 °C for 10 minutes for immunoblotting.

Quantitative Real-time PCR (qRT-PCR)

Total RNA was extracted from mouse glomeruli using RNeasy Mini Kit (QIAGEN, 74104) according to manufacturer's instructions. 600 ng of total RNA was reverse transcribed into cDNA using the mix of Thermo Scientific RevertAid Reverse Transcriptase (EP0442), dNTP mix (Thermo Scientific, R0192), random hexamer primer (Thermo Scientific, SO142), Thermo Scientific Ribolock RNase (EO0381) and reaction buffer following the instructions. Quantitative real-time PCR (qRT-PCR) was carried out using a QuantStudio 3 Real-Time PCR system (Applied Biosystems) and the Power up SYBR Green PCR Master Mix (Applied Biosystems). The level of specific mRNA was normalized to 18s expression. The relative mRNA expression was determined by the $2^{-\Delta\Delta Ct}$ method. Samples were assayed in duplicates. The sequences of primers were: Egr1 forward TATGAGCACCTGACCACAGAG, Egr1 reverse GCTGGGATAACTCGTCTCCA; Egr3 forward CCGGTGACCATGAGCAGTTT, Egr3 reverse TAATGGGCTACCGAGTCGCT; Tnfrsf12a forward GTGTTGGGATTCGGCTTGGT, Tnfrsf12a reverse GTCCATGCACTTGTCGAGGTC; Tnfaip3 forward AAGCTCGTGGCTCTGAAAACC, reverse CCGAGTGTCGTAGCAAAGTCCT; 18s forward CACGGCCGGTACAGTGAAAC, reverse AGAGGAGCGAGCGACCAAA.

Proteomics sample preparation

Isolated mouse glomeruli were taken up in 6 M GuaHCl, 0.1 M HEPES, 5 mM EDTA, 1x Halt Protease and Phosphatase Inhibitor Cocktail (Thermo Scientific). Samples were homogenised at 30 Hz for 1 minute using a bead mill, sonicated to shear DNA, and centrifuged for 5 minutes at 13,000 g to remove insoluble debris. The supernatant was transferred to a fresh tube and the protein concentration was determined using a commercial BCA kit (Thermo Scientific). Protein aliquots of 50 µg were used for sample preparation. For co-immunoprecipitation experiments, the eluate was adjusted to a final concentration of 5% SDS in PBS (pH 7.4). All samples were reduced with 10 mM tris(2-carboxyethyl)phosphine (TCEP) and alkylated using 50 mM chloroacetamide (CAA) at 95 °C for 5 minutes. Overnight digestion was carried out using trypsin (SERVA) at 1:50 enzyme to substrate ratio at 37 °C. Tryptic peptides were purified/desalted using in-house STAGE-Tips (Affinisep SPE-Disks-Bio-C18-100.47.20) or using magnetic beads (SeraMag Cytiva 65152105050250 and 45152105050250). Purified peptide samples were separated on an Ultimate3000 RSLC nano-high performance liquid chromatography (HPLC) coupled on-line to an Exploris480 orbitrap tandem mass spectrometer (Thermo Scientific). The HPLC was operated in a two-column setup with an Acclaim 5mm C18 cartridge pre-column (Thermo Scientific) and an ionOpticks Aurora Ultimate 25 cm column (3rd generation) with integrated emitter tip. Separation was performed at 400 nL/minute in a heated column oven at 50 °C (Sonation) with the following gradient of solvents A (H_2O + 0.1% FA) and B (ACN + 0.1% FA): 5 minutes from 2-8% B, 80 minutes from 8-25% B, 10 minutes from 25-35% B and a high-organic washout at 90% B for 8 minutes followed by a re-equilibration to the starting conditions (2% B). The mass spectrometer was operated with a field asymmetric ion mobility spectrometry (FAIMS) device at standard resolution with a total carrier gas flow of 3.8 L/minute at two compensation voltages (CVs): -45 and -65V. The Orbitrap resolution for the MS1 full scan was set to 120k, whereas the MS2 DIA scans were recorded with 15 isolation windows from 400-1000 m/z for both FAIMS CVs at an orbitrap resolution of 30k.

Analysis of microarray data

Microarray profiles from glomerular compartment of kidney biopsies from MN patients and healthy controls in the NEPTUNE cohort (GSE200828) were retrieved from Gene Expression Omnibus (GEO) repository. Differential expression analysis was performed using R package limma (v3.54.2) (11), with thresholds of an absolute $\log_2$ fold change (FC) > 0.5 and Benjamini-Hochberg adjusted P-value < 0.05.

Analysis of single cell RNA-seq datasets

Glomerular single-cell RNA sequencing (scRNA-seq) datasets GSE146912 were retrieved from GEO. For quality control, we included only genes detected in more than 3 cells and retained cells between 200 and 7500 genes. Cells with over 10% of reads mapping to mitochondrial genes or ribosomal genes were excluded. Data processing and clustering were performed using R package Seurat (version 4.3.0.1) (12). Data was normalized using the NormalizeData function, followed by identification of 2000 highly variable genes and data scaling using ScaleData function. Principal component analysis (PCA) was conducted using the RunPCA function, and significant principal components were determined based on ElbowPlot. Unsupervised clustering was performed using the FindNeighbors and FindClusters functions with default parameters and a resolution of 0.2. Batch effect across samples were corrected using Harmony (13). Uniform Manifold Approximation and Projection (UMAP) was then plotted to visualize clustering results. Major glomerular cell types podocytes, mesangial cells and glomerular endothelial cells were annotated based on known marker genes. Heatmaps showing the relative expression of selected genes was generated after scaling the average expression levels in podocytes, mesangial cells and glomerular endothelial cells.

Supplementary References

1. Skarnes WC, et al. A conditional knockout resource for the genome-wide study of mouse gene function. *Nature.* 2011;474(7351):337-342.
2. Köllner SMS. Antigen-specific therapies of membranous nephropathy. *Doctoral dissertation*. Universitaet Hamburg (Germany); 2023. https://ediss.sub.uni-hamburg.de/handle/ediss/10250.
3. Grahammer F, et al. A flexible, multilayered protein scaffold maintains the slit in between glomerular podocytes. *JCI Insight.* 2016;1(9):e86177.
4. van den Berg JG, et al. Podocyte foot process effacement is not correlated with the level of proteinuria in human glomerulopathies. *Kidney Int.* 2004;66(5):1901-1906.
5. Gang NF. A Rapid Method for the Isolation of Glomeruli from the Human Kidney. *American Journal of Clinical Pathology.* 1970;53(2):267-269.
6. Tomas NM, et al. Thrombospondin type-1 domain-containing 7A in idiopathic membranous nephropathy. *N Engl J Med.* 2014;371(24):2277-2287.
7. Bochel A, et al. Structure of the N-terminal didomain d1_d2 of the Thrombospondin type-1 domain-containing 7A. https://doi.org/10.1101/2023.05.03.539264. Posted on bioRvix May 03, 2023.
8. Seifert L, et al. The most N-terminal region of THSD7A is the predominant target for autoimmunity in THSD7A-associated membranous nephropathy. *J Am Soc Nephrol.* 2018;29(5):1536-1548.
9. Seifert L, et al. The classical pathway triggers pathogenic complement activation in membranous nephropathy. *Nat Commun.* 2023;14(1):473.
10. Zhang J, et al. Production of chimeric heavy-chain antibodies. *Methods Mol Biol.* 2009;525:323-336, xv.
11. Ritchie ME, et al. limma powers differential expression analyses for RNA-sequencing and microarray studies. *Nucleic Acids Res.* 2015;43(7):e47.
12. Hao Y, et al. Integrated analysis of multimodal single-cell data. *Cell.* 2021;184(13):3573-3587.e3529.
13. Korsunsky I, et al. Fast, sensitive and accurate integration of single-cell data with Harmony. *Nat Methods.* 2019;16(12):1289-1296.

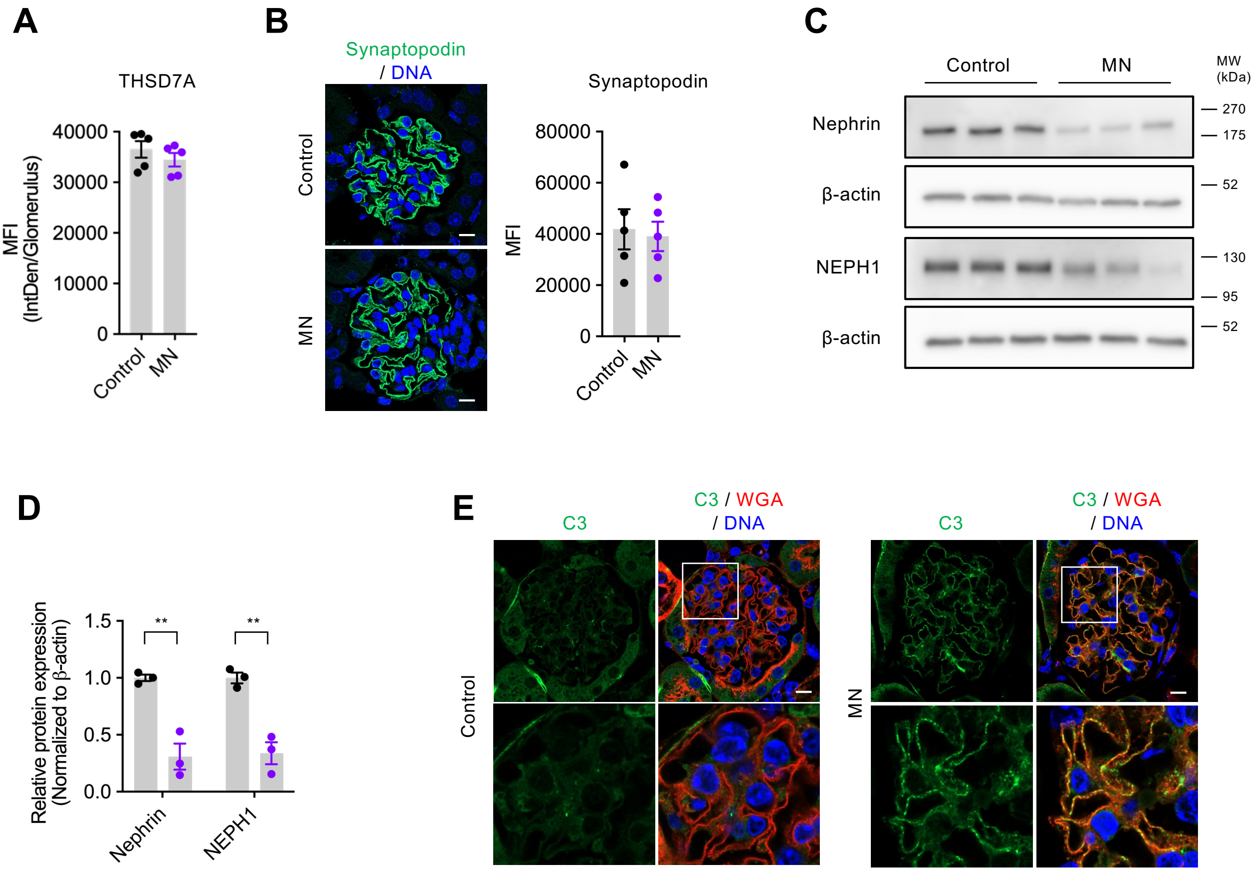

Supplementary Figure 1. Induction of glomerular injury in experimental THSD7A-associated membranous nephropathy (MN). (**A**) Quantification of mean fluorescence intensity (MFI) of THSD7A immunofluorescence staining, n=5 animals per group. (**B**) Immunofluorescence stainings for synaptopodin (green) in mice injected with control IgG or anti-THSD7A IgG. Nuclei were counterstained with Hoechst (blue). Bar = 10 μm. Quantification of MFI of synaptopodin per glomerulus tuft is shown in right panel, n=5 animals per group. (**C**) Immunoblotting images showing the reduced expression of nephrin and NEPH1 in isolated glomeruli of MN mice. MW, molecular weight. (**D**) Individual protein intensity was quantified by densitometry, and plotted over the intensity of β-actin. ***P* <0.01 (unpaired t-test). Data are presented as mean $\pm$ SEM, n=3. (**E**) Representative immunofluorescence stainings for complement C3 (green) and wheat germ agglutinin (WGA, red) in mice injected with control IgG (left panel) or anti-THSD7A IgG (right panel). Nuclei were counterstained with Hoechst (blue). Bar = 10 μm.

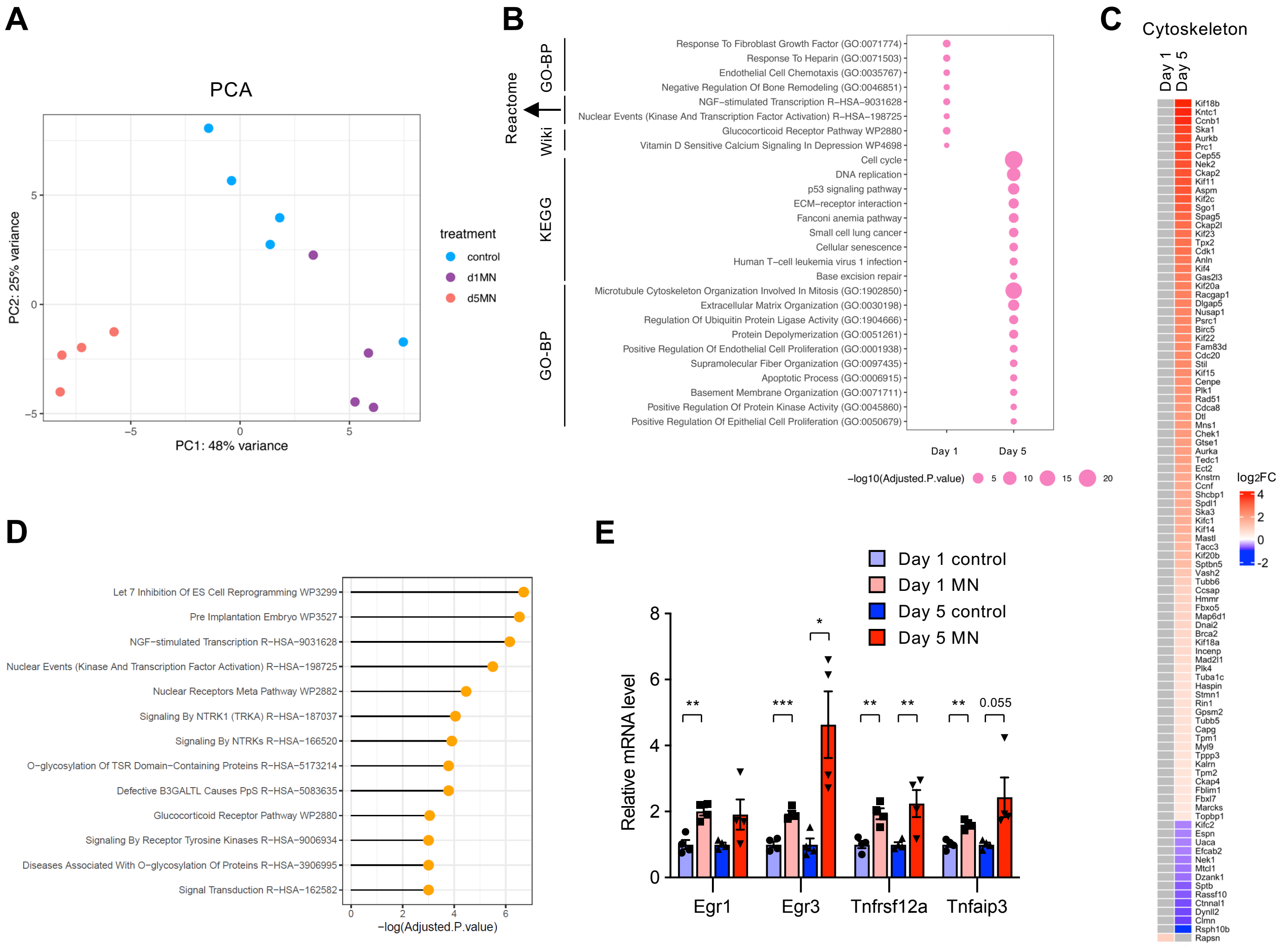

Supplementary Figure 2. Transcriptomic profiles in experimental THSD7A-associated MN. (**A**) Principal component analysis (PCA) plot for the glomerular samples used for bulk RNA-seq. (**B**) Pathway enrichment analysis of differentially expressed genes from bulk RNA-seq at day 1 or day 5 MN vs. control, performed using Enrichr. The plot shows the significantly activated pathways. (**C**) Heatmaps demonstrating the log_2 fold change (FC) of differentially expressed genes (DEGs) of day 1 or day 5 MN vs. control for cytoskeleton markers. Nonsignificant genes are shown in grey. (**D**) Pathway enrichment of the common genes in comparisons of day 1 vs. control and day 5 MN vs. control from bulk RNA-seq, performed by Enrichr. Only significant pathways (adjusted *P*-value <0.05) are shown. (**E**) mRNA expression of several DEGs identified in bulk RNA-seq analysis, as assessed by qRT-PCR in isolated glomeruli from day 1 and day 5 MN and control mice. $^*P < 0.05$, $^{**}P < 0.01$, $^{***}P < 0.001$ (unpaired t-test). Data are presented as mean ± SEM, n=4.

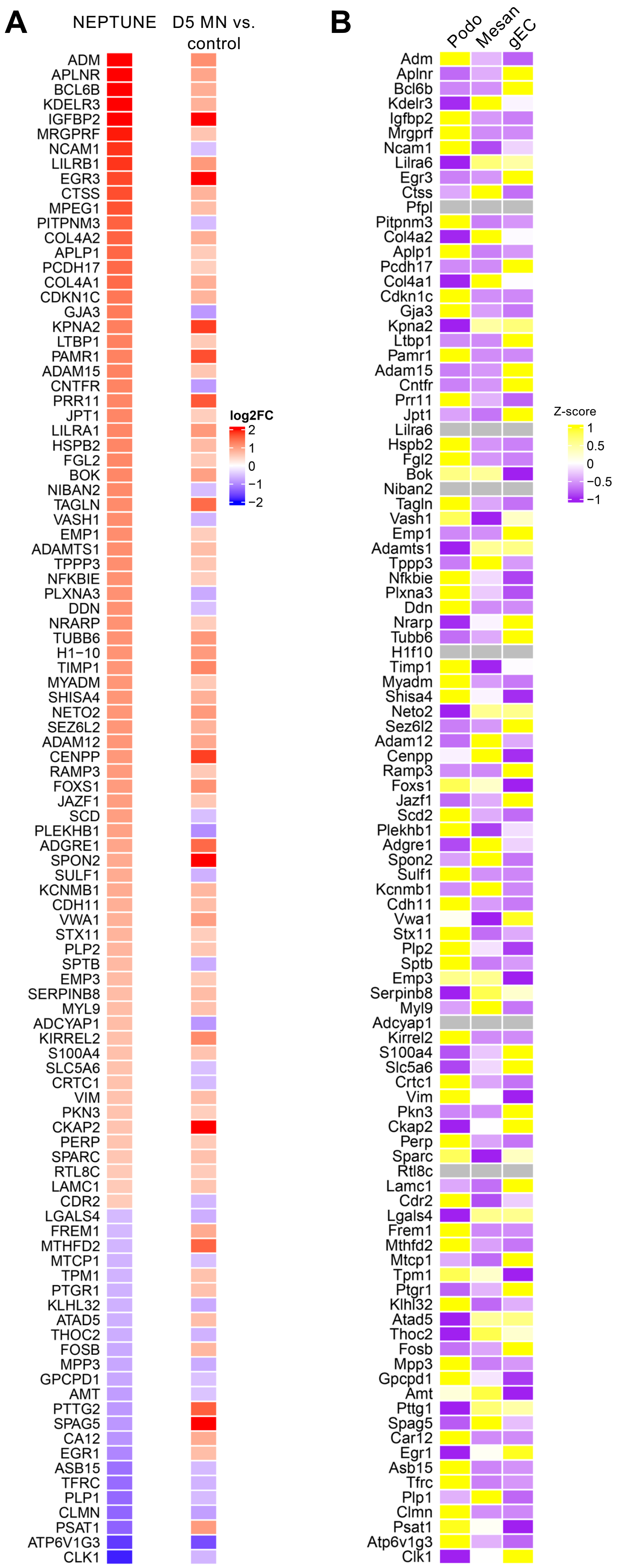

Supplementary Figure 3. Mapping of differentially expressed genes (DEGs) in MN models to transcriptomic profiles of MN patients. (**A**) Heatmap displaying common DEGs identified in glomeruli from day 5 MN mouse model and MN patients (glomerular microarray data from NEPTUNE cohort, GSE200828). The color range represents the log_2FC by comparison of day 5 MN model vs. control, or MN patients vs. healthy control. (**B**) Heatmap showing the relative expression (Z-score) of common DEGs from (A) across major glomerular cell types in a single-cell RNA-seq dataset (PMID: 32651223). Genes not detected in scRNA-seq dataset are shown in grey. Podo: podocytes, Mesan: mesangial cells, gEC: glomerular endothelial cells.

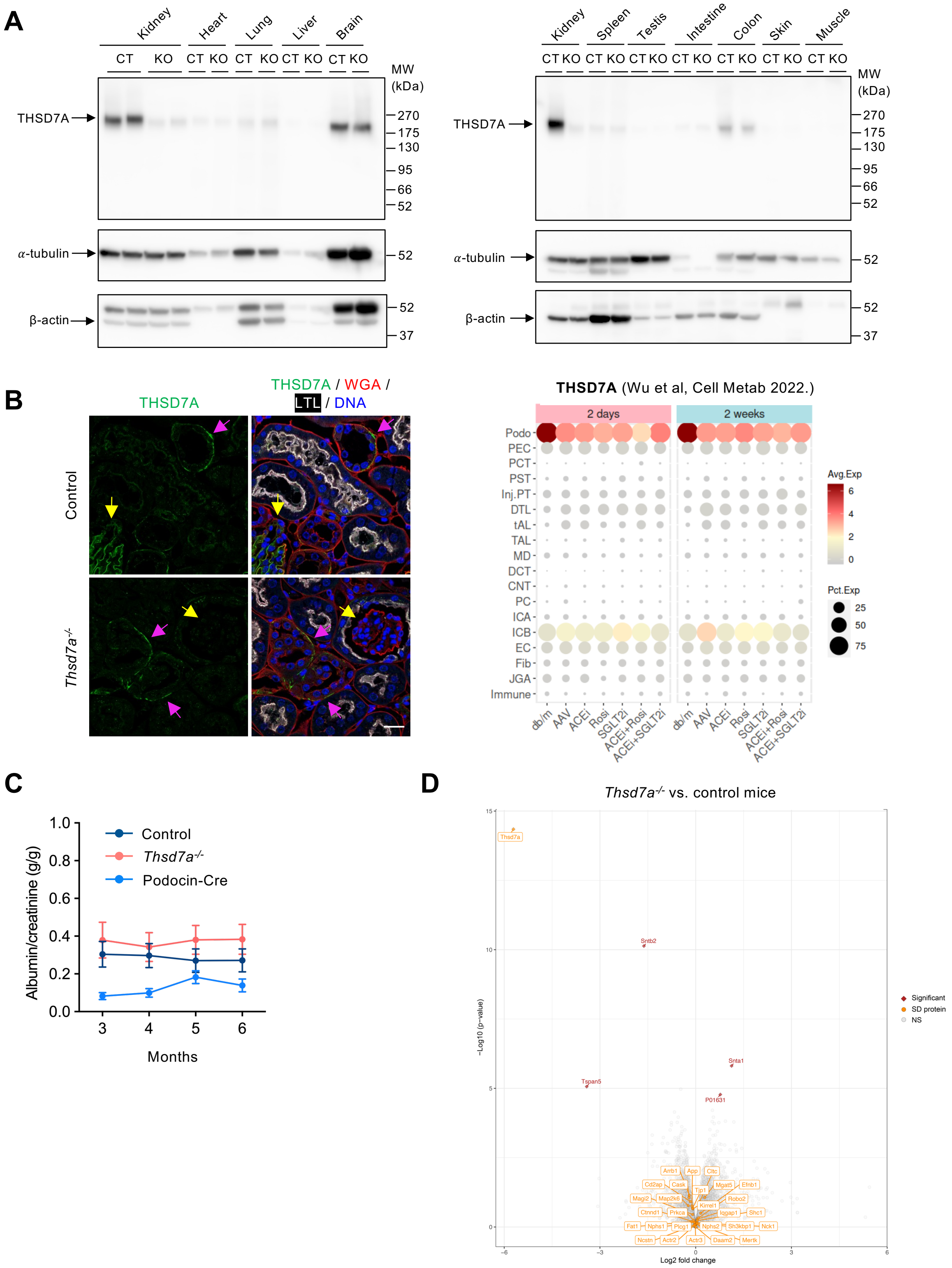

Supplementary Figure 4. Basal characterization of *Thsd7a*$^{-/-}$ mice. (**A**) Immunoblotting analysis for THSD7A levels in different organ lysates from control (CT) and *Thsd7a*$^{-/-}$ mice. α-tubulin and β-actin were used as internal control proteins. (**B**) Representative immunofluorescence staining for THSD7A (green), WGA (red) and LTL (white, a marker of proximal tubules) in control and *Thsd7a*$^{-/-}$ mice (left panel). Pink arrows point at expression of THSD7A in tubules, and yellow arrows indicate the glomeruli (bar = 20 μm). Dot plot showing the relative *Thsd7a* expression in single nucleus RNA-seq (snRNA-seq) datasets from kidneys of diabetic kidney disease mouse models and control mice (Wu et al, Cell Metab 2022, plot generated from website https://humphreyslab.com/SingleCell/) (right panel). (**C**) Albuminuria as measured by albumin-to-creatinine ratio (g/g) from the age of 3 to 6 months in control, *Thsd7a*$^{-/-}$ and podocin-Cre mice. Data are presented as mean ± SEM, n=22-23 in control and *Thsd7a*$^{-/-}$ group (same values as in Figure 2E), n=7-8 in podocin-Cre group. (**D**) Volcano plot of proteomic differential expression analysis generated from isolated glomeruli of control and *Thsd7a*$^{-/-}$ mice (n=6 per group). X-axis represents the $\log_2$ fold change (FC) of label-free quantification intensity of proteins comparing *Thsd7a*$^{-/-}$ with control mice, and y-axis indicates the -$\log_{10}$ *P*-value. Red colored proteins meet the significance threshold (limma moderated t-test, BH-adjusted *P*-value < 0.05, $|\log_2 FC| > 0.5$), and slit diaphragm proteins are highlighted by orange color dots.

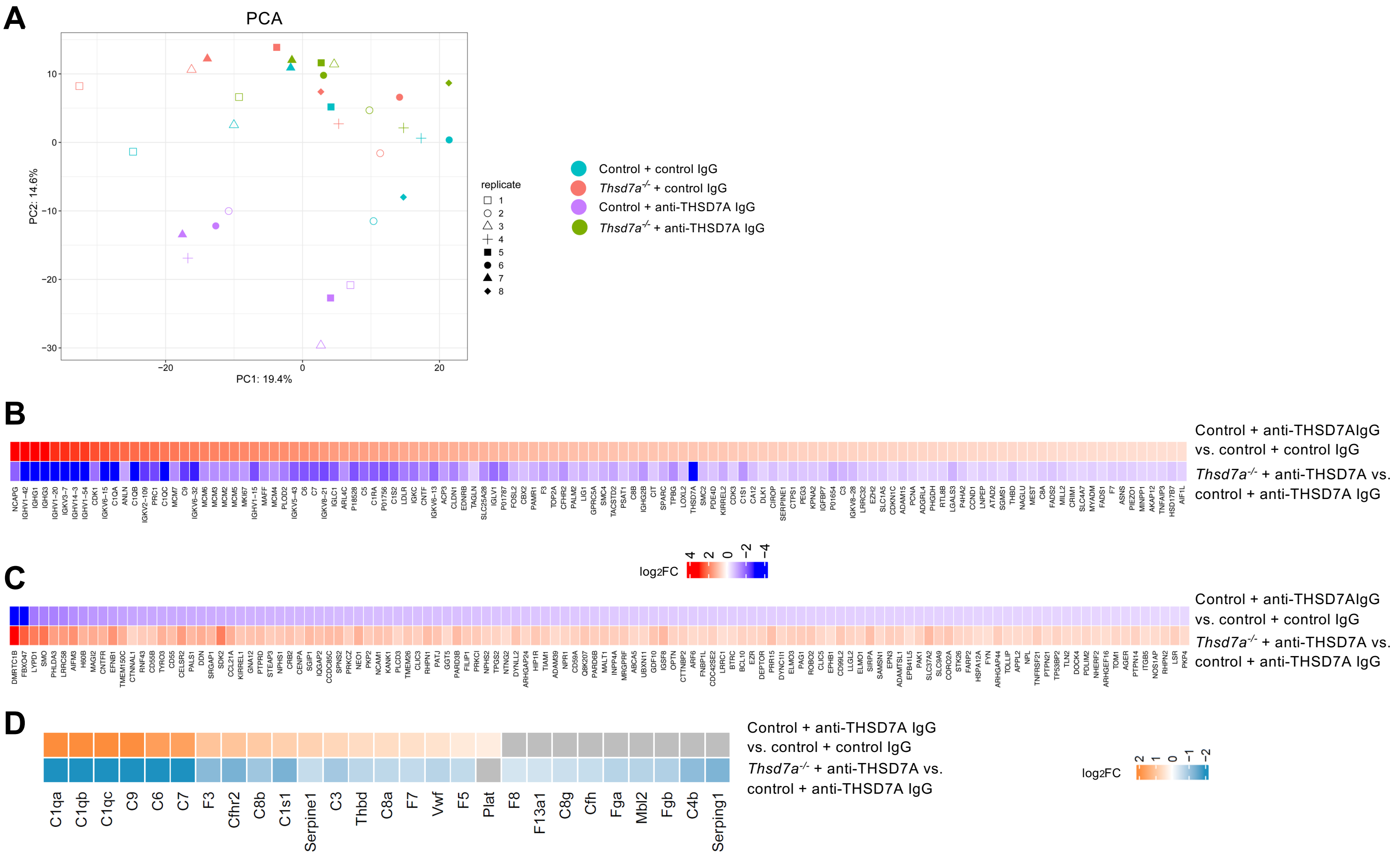

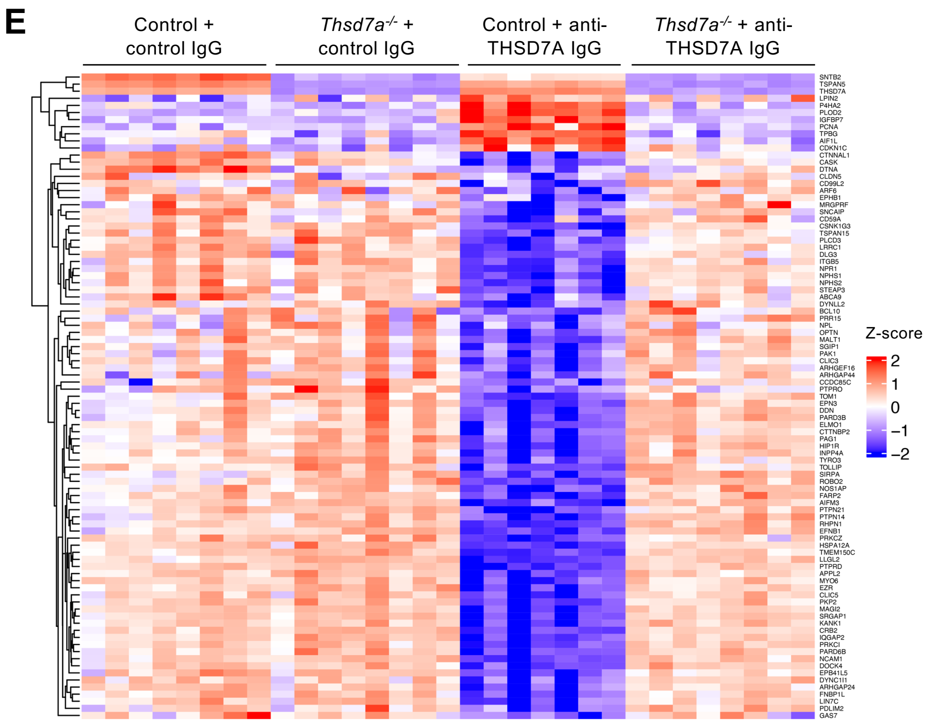

Supplementary Figure 5. Proteomic analysis of experimental THSD7A-associated MN. (**A**) Principal component analysis (PCA) of proteome from control and *Thsd7a*$^{-/-}$ mice treated with control or anti-THSD7A IgG (correspondent with Figure 4). (**B**) Heatmap revealing the $\log_2$ FC of significant proteins that were upregulated in control mice + anti-THSD7A IgG group (control mice + anti-THSD7A IgG vs. control mice + control IgG) and downregulated when THSD7A is deficient (*Thsd7a*$^{-/-}$ mice + anti-THSD7A IgG vs. control mice + anti-THSD7A IgG group). (**C**) Heatmap showing $\log_2$FC of the significant expressed proteins that were downregulated in control MN mice (control mice + anti-THSD7A IgG vs. control mice + control IgG) and upregulated in *Thsd7a*$^{-/-}$ MN mice (*Thsd7a*$^{-/-}$ mice + anti-THSD7A IgG vs. control mice + anti-THSD7A IgG). (**D**) Heatmap representing $\log_2$ FC of significantly altered proteins in the complement signaling pathway. Data are from proteomic comparisons of control mice + anti-THSD7A IgG vs. control mice + control IgG, and *Thsd7a*$^{-/-}$ + anti-THSD7A IgG vs. control + anti-THSD7A IgG group. (**E**) Heatmap showing the relative protein expression (Z-score) of podocyte specific proteins in proteome of control and *Thsd7a*$^{-/-}$ mice treated with control or anti-THSD7A IgG.

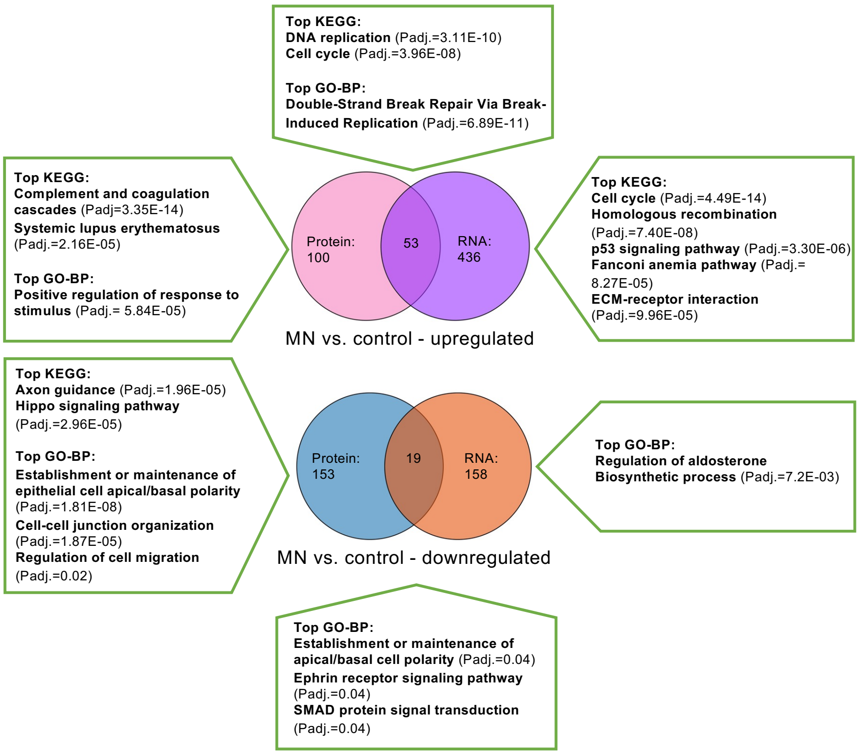

Supplementary Figure 6. Integrated analysis of transcriptome and proteome. Venn diagrams showing the overlap and distinction of upregulated (top panel) and downregulated (bottom panel) differentially expressed genes and proteins between transcriptomic and proteomic datasets, and pathway enrichment analysis of the common and unique genes using Enrichr.

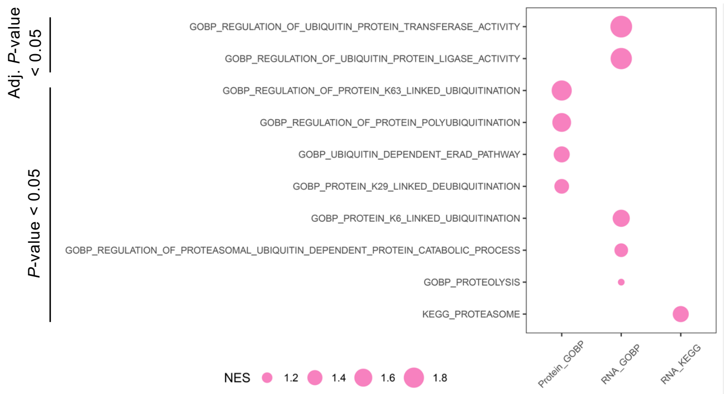

Supplementary Figure 7. Proteolysis pathway enrichment. Functional enrichment analysis in transcriptomics and proteomic datasets using fgsea reveals significantly enriched protein degradation pathways in MN vs. control mice (adjusted *P*-value < 0.05 or *P*-value as indicated relatively).

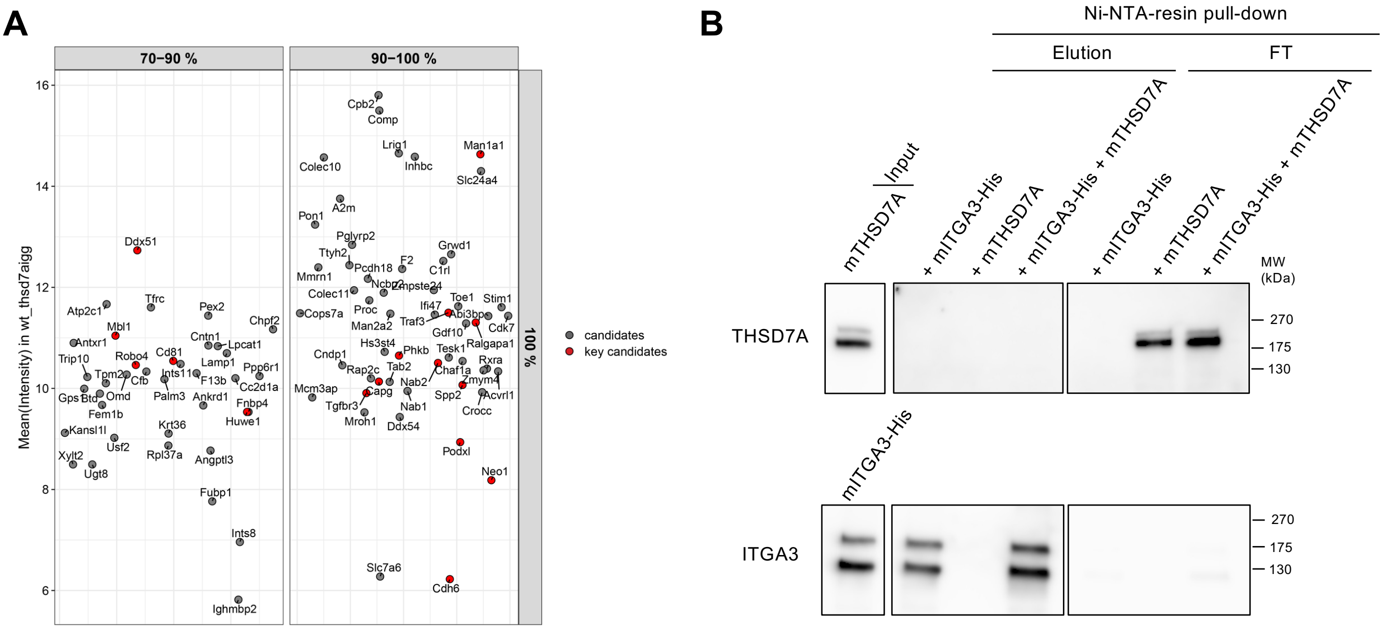

Supplementary Figure 8. Coimmunoprecipitation (co-IP) and pull-down experiments showing the indirect interaction of THSD7A and ITGA3. (A) Extension plot for possible THSD7A interactors generated by co-IP/MS from mouse glomeruli, including proteins which are present in over 70% of THSD7A-IgG co-IP samples, while absent in 100% of control IgG co-IP samples. Y axis indicates the mean intensity of each protein in THSD7A-IgG co-IP group, and the key candidates were highlighted by red color. (**B**) Pull-down experiments. Recombinant His-tagged mouse integrin α3β1 (mITGA3-His) was immobilized on Ni-NTA resin and incubated with or without recombinant mouse THSD7A extracellular domain (mTHSD7A). Bound proteins were eluted and immunoblotted with the indicated antibodies. Input lanes contain 50ng of each protein used in the assay. Flow-through (FT) lanes contain 5% of the unbound fraction.
